# Consensus in the weighted voter model with noise-free and noisy observations

**DOI:** 10.1007/s11721-025-00248-z

**Published:** 2025-05-06

**Authors:** Ayalvadi Ganesh, Sabine Hauert, Emma Valla

**Affiliations:** 1 School of Mathematics, University of Bristol, Bristol, BS8 1UG UK; 2 School of Engineering Mathematics and Technology, University of Bristol, Bristol, UK

**Keywords:** Consensus, Weighted voter model, Decentralised decision-making

## Abstract

Collective decision-making is an important problem in swarm robotics arising in many different contexts and applications. The Weighted Voter Model has been proposed to collectively solve the best-of-*n* problem, and analysed in the thermodynamic limit. We present an exact finite-population analysis of the best-of-two model on complete as well as regular network topologies. We also present a novel analysis of this model when agent evaluations of options suffer from measurement error. Our analytical results allow us to predict the expected outcome of best-of-two decision-making on a swarm system without having to do extensive simulations or numerical computations. We show that the error probability of reaching consensus on a suboptimal solution is bounded away from 1 even if only a single agent is initialised with the better option, irrespective of the total number of agents. Moreover, the error probability tends to zero if the number of agents initialised with the best solution tends to infinity, however slowly compared to the total number of agents. Finally, we present bounds and approximations for the best-of-*n* problem.

## Introduction

Robot swarms are typically composed of large numbers of simple, low-cost robots. Their use has been proposed in a variety of applications ranging from autonomous agriculture (Blender et al., 2016) and environmental monitoring (Jeradi et al., 2015), to the exploration of disaster sites (Hauert et al., 2009). Robots in a swarm operate by reacting to their local environment or neighbouring robots. While the sensing and cognitive abilities of individual robots may be limited, the swarm nevertheless needs to achieve effective coordinated action (Hamann, 2018). This requires the development of decentralized control mechanisms which enable the swarm to be scalable, adaptable, and robust to failures of robots. Many coordination tasks can be captured by the abstraction of *collective decision-making*. In this paper, we analyse a decentralized algorithm for one such task, known as the best-of-*n* problem, described below.

Collective decision-making is relevant to a class of problems in which all or most agents need to converge on one choice amongst many (Brambilla et al., 2013). It has been observed in living organisms such as honeybees deciding on nest sites (Franks et al., 2002) and birds moving as a flock (Okubo, 1986). Best-of-*n* problems are a subset of collective decision-making problems where the choice is from a finite set of *n* options, as opposed to a continuous range. They are differentiated on the basis of whether the choice is between identical options, or options differing in quality or cost. The objective is to develop simple, decentralized algorithms which enable the agents to reach consensus on the best alternative (or any one if the options are identical), and to do so quickly. Design approaches to best-of-*n* problems can be categorized as either ‘bottom-up’ or ‘top-down’ (Valentini, 2017). Bottom-up approaches may be further divided into opinion-based methods, where agents communicate explicitly with each other, and environmentally based methods where information is inferred from agent actions.

In this paper, we focus on one particular opinion-based method for solving the best-of-*n* problem, known as the Weighted Voter Model (Valentini et al., 2014), which was inspired by house-hunting honeybee swarms and is described in greater detail below. We follow (Valentini et al., 2014) and focus on a best-of-two problem with alternatives *A* and *B*, of which *A* is assumed to be better. Success is defined as all agents reaching consensus on option *A*. While we only present rigorous results for the best-of-two problem, we also present bounds and heuristics for the best-of-*n* problem and compare them with simulations.

The main contribution of our analysis is that it allows us to predict the expected outcome of the Weighted Voter Model without the need for simulations or extensive numerical computations; hence, its complexity does not grow with the size of the swarm. We believe that the techniques introduced in this paper can be extended to other swarm algorithms, but this is a topic for future research. In this paper, we present exact finite-population predictions for the probability of reaching consensus on the best option in the best-of-two problem on complete graphs or regular graphs; these are given in Theorem 1 in Sect. 2.1, while a bound on the consensus time is given in Theorem 2 in Sect. 2.2. We also present bounds and approximations for the best-of-*n* problem (Corollary 1 and Conjecture 1 in Sect. 2.1), and for graphs which are only approximately regular (Theorem 3 in Sect. 2.3), and demonstrate that these are supported by simulations. We further extend the analysis to scenarios in which agent measurements of site qualities are corrupted by noise; expressions for the consensus probability in large systems are given in Theorem 5 in Sect. 3, assuming Conjecture 2 is true. Simulation results support the predictions.

### The weighted voter model

The Weighted Voter Model sets the best-of-two consensus problem in an environment that includes three regions, the ‘nest’, and sites *A* and *B*. Agents start off in the nest and are initialized with arbitrary preferences, which we interchangeably refer to as opinions. Whenever an opinion is initialized or updated, the agents leave the nest to survey the site that corresponds to their opinion and measure its quality with their sensory apparatus. They then return to the nest site, and advertise their opinion for a time that is exponentially distributed with mean equal to the measured quality. At the end of this period, when the agent has stopped signalling their opinion, they adopt the opinion being advertised by a randomly chosen neighbour. The correlation between the measured site quality and the length of time for which it is advertised by an agent introduces a positive feedback mechanism which boosts the chances of reaching consensus on the better site. We now specify the model formally.

Consider a population of *N* agents, which we identify with the nodes or vertices of a connected graph $G=(V,E)$ with vertex set *V* and edge set *E*. The edges specify which pairs of agents can communicate directly. We assume that edges are undirected, i.e., $(u,v)\in E$ if and only if $(v,u)\in E$. By the neighbourhood of a node $v$, we mean the set of nodes to which it has edges, i.e., $\{ u:(u,v)\in E \}$; the cardinality of this set is called the degree of $v$, denoted *deg*($v$). The graph *G* is called *complete* if it contains all possible edges, i.e., if $E=V\times V$. It is called *d*-*regular* if all nodes have the same degree, *d*.

The agents seek to reach consensus on the better of two options, *A* and *B*, of differing quality. The qualities are represented by real numbers, $q_A>q_B>0$. At any time $t\ge 0$, each agent $v\in V$ has a preference for one of the sites, which we denote by $X_v(t)\in \{ A,B \}$. The algorithm starts from arbitrary initial values for the opinions of agents. Whenever the opinion of an agent $v$ is initialized or updated, the agent samples the option that corresponds to their opinion and obtains a measure $q_v>0$ of its quality. If the measurement is accurate, then $q_v=q_A$ or $q_B$ depending on *v*’s opinion. We allow for the possibility that the measurement is noisy. The process of sampling an option and measuring its quality is assumed to be instantaneous. Once agent $v$ has obtained a measurement $q_v$ of the quality of its preferred option, it retains that preference for a random length of time which has an exponential distribution with parameter $1/q_v$ (denoted $Exp(1/q_v)$); the mean of this random variable is $q_v$. We assume that the random variables corresponding to different agents, and different measurements taken by the same agent, are mutually independent. At the end of this period, the agent relinquishes its opinion, chooses an agent *w* uniformly at random from among its neighbours (namely, nodes *u* such that $(u,v)\in E$) and adopts the opinion of *w*. It then repeats the process of sampling the site corresponding to that opinion, even if the opinion did not change, and the process continues. The process of contacting a neighbour, adopting its opinion, sampling the associated site and estimating its value is assumed to be instantaneous.

The algorithm described above involves some idealizations. In practice, sampling an option and measuring its quality takes time. But, if the times between opinion updates by an agent are large compared to the time required for measurement, then the idealization is justifiable. Secondly, we assume the network remains unchanging over time. This is unrealistic for many swarm robotics applications. It may be satisfied (over the time scale needed to reach consensus) in applications in which robots can assess the quality of an option without moving from their current location; see, e.g., (Ebert et al., 2020; Shan & Mostaghim, 2021). Besides, evolving networks tend towards better satisfying the *well-mixed population* assumption. Hence, we conjecture that the bounds and guarantees provided by our static network analysis continue to hold for networks evolving independently of the opinions. We present simulation results supporting this conjecture. The main quantities of interest in the algorithm are the probability of reaching consensus on the better option, *A*, and the time required to do so. We derive expressions for these quantities.

### Related work

The Weighted Voter Model takes inspiration from collective decision-making strategies observed in human and primate groups (Conradt & List, 2009; Couzin et al., 2011), and insect colonies (Marshall et al., 2009; Kao et al., 2014). Other sources of inspiration include theoretical frameworks for opinion dynamics in statistical physics (Castellano et al., 2009). It has been analysed using a variety of methods, including ordinary differential equations (o.d.e.s), Markov chains, and agent-based simulations. We now describe these in more detail.

Theoretical analyses of the Weighted Voter Model have typically assumed a large number of well-mixed agents, thereby justifying a mean-field approach. There have then been two different approaches to the study of the mean-field approximation. One is to model it by a system of ordinary differential equations (o.d.e.s) (Montes de Oca et al., 2011), which can be solved to obtain the limit point to which the system converges (Lambiotte et al., 2009). This approach yields deterministic models and is only valid in the large population limit. Another approach is to incorporate randomness and finite-size effects using Markov Chains; see, e.g., (Valentini et al., 2013; Hamann, 2013). Here, the proportion of agents in different states evolves as a Markov process. This approach is used to quantify the effect of finite swarm size on consensus probabilities. It does not have a spatial element either, and relies on a ‘well-mixed’ assumption of agent interaction. Finally, agent-based models incorporate both stochastic and spatial elements by representing the robots as agents performing random walks on simple network structures such as a 1D or 2D lattice (Lambiotte et al., 2009). All three of these approaches are compared in Valentini et al. (2014).

Markov process models of the Weighted Voter Model have mostly either been solved numerically, or simulated using the Gillespie algorithm (Gillespie, 1977). There has been little work on obtaining closed-form bounds or approximation on consensus probabilities and times until the recent work of Mukhopadhyay et al. (2020). In this paper, we extend and build upon their work. Finally, connections have been established between the network structure of interacting agents and the speed of information diffusion (Olfati-Saber et al., 2007), as well as between the rate of convergence of consensus algorithm and the algebraic connectivity of the network (Hatano & Mesbahi, 2005). We present consensus time bounds involving isoperimetric constants of the interaction network.

### Our contributions

The motivation for this work is to predict the outcome of a swarm system analytically, without the need either for computationally expensive simulations or numerical computations. To this end, we present a rigorous mathematical analysis of the Weighted Voter Model with only two options. We first assume that assessments of site quality are error-free, so that $q_v=q_A$ or $q_B$. It then follows from the verbal description above that the vector of agent opinions, denoted ${\textbf{X}}(t)=(X_v(t), v\in V)$, evolves as a Markov process on the state space $\{ A,B \}^V$, and that reaching consensus on *A* corresponds to the Markov process hitting the all-*A* state before the all-*B* state.

Our first main contribution is to derive exact analytical expressions for the probability of reaching consensus on *A*, and bounds on the time to reach consensus, when the communication graph *G* is regular (all vertices have the same degree) and connected. While the result on consensus probability appears not to be well-known in the robotics community, it is not new; the Weighted Voter Model is the same as the ‘biased voter model’ studied in Mukhopadhyay et al. (2020), and the Moran model with selection,^1^ which has been analyzed in Durrett (2008). We include the analysis for completeness, and because it clarifies the analysis of models in which the communication graph is only approximately regular. The bound on consensus time is new to the best of our knowledge. Additionally, we obtain bounds on consensus probabilities and times when the graph *G* is only “approximately” regular. This analysis is inspired by Adlam and Nowak (2014), but not identical to it. While we only derive exact analytical results for the best-of-two problem, we also provide bounds and heuristics for the best-of-*n* problem, and compare these to simulations.

The second major contribution of this paper is an analysis of the Weighted Voter Model with noisy measurements of site quality, albeit only on a complete graph. We conjecture that the consensus probability in this setting is well approximated by the extinction probability of a related multi-type branching process. We show that this extinction probability is the solution of a fixed-point equation, and present a numerical procedure which is guaranteed to solve it. Finally, we present simulation results for all the models studied, which we compare with theoretical predictions and bounds.

In addition to exact expressions, our analysis provides some qualitative insights about consensus probabilities. It shows that, even if only a single agent initially prefers the best option, then the probability of reaching consensus on this option is bounded below by a positive constant, which only depends on the ratio of qualities of the two options and not on the number of agents. Secondly, the error probability of reaching consensus on the worse option decays exponentially in the number of agents initialised with the best option, with the error exponent depending on the ratio of site qualities. These insights suggest that the Weighted Voter Model is a highly robust mechanism for the best-of-two problem. Finally, we present a bound and a heuristic for the best-of-*n* problem, while deferring a full, rigorous analysis of this more general setting to future work.

The rest of the paper is organised as follows. In Sect. 2, we present an exact finite-population analysis of the model when agents measure site qualities without error. In Sect. 3, we propose a heuristic for analysing the model when site quality measurements are imperfect. We compare the theoretical analysis in these two chapters with Monte Carlo simulations in Sect. 4 before concluding in Sect. 5.

## Consensus with noise-free measurements

In this section, we calculate the probability of reaching consensus on the worse option, *B*, as a function of the initial condition. We also bound the time to reach consensus on either option. Site quality assessments by agents are assumed to be error-free. Hence, an agent whose opinion has been initialized or updated to *A* will signal that opinion for an $Exp(1/q_A)$ random time for updating its opinion. Likewise, opinion *B* will be signalled for an $Exp(1/q_B)$ random time before being updated. We set $q_A=1$ without loss of generality (w.l.o.g.) as this simply corresponds to a choice of the units in which time is measured. It will be notationally convenient to denote $1/q_B$ by $\lambda$. Since $q_B<q_A$, it follows that $\lambda >1$.

Thus, we have a Weighted Voter Model in which preferences for *A* are updated at rate 1, i.e., after *Exp*(1) random times, and preferences for *B* at rate $\lambda >1$, i.e., after $Exp(\lambda )$ random times. Equivalently, we may assume that there are two independent Poisson processes at each node, of rates or intensities 1 and $\lambda$, and independent of the Poisson processes at other nodes. If a node has opinion *A* (respectively, *B*), then it updates its opinion when there is an increment of the rate 1 (resp., rate $\lambda$) Poisson process at that node. It does so by contacting a neighbour chosen uniformly at random, and adopting the opinion of that neighbour.

Rather than directly analysing the above continuous-time Markov process, it will be more convenient to work with the embedded jump chain, which we now define, first in the general case and then for our specific model. We also recall some facts about Markov chains that will be useful in the sequel; see, e.g., Norris (1997) for further details. Let $X(t), t\in {\mathbb {R}}_+$ be a continuous-time Markov chain on a finite state space ${\mathcal {X}}$. Let $q_{xy}, x,y\in {\mathcal {X}}$, $x\ne y$, denote the transition rates of *X*(*t*), i.e., ${\mathbb {P}}(X(t+dt)=y| X(t)=x)= q_{xy}dt+o(dt)$. For $i\in {\mathbb {N}}$, define $T_i$ to be the $i^\textrm{th}$ jump time of *X*(*t*), namely the $i^\textrm{th}$ time that it changes state. Define $Y(i)=X(T_i)$ to be the state of the Markov chain immediately *after* the $i^\textrm{th}$ jump. (More formally, it is conventional to define the sample paths of *X*(*t*) to be right-continuous.) Then, conditional on $Y(i)=x\in {\mathcal {X}}$, the random time interval $T_{i+1}-T_i$ until the next jump of *X*(*t*) has an $Exp(q_x)$ distribution, where $q_x=\sum _{z\ne x} q_{xz}$ is the total jump rate out of state *x*. Moreover, $Y(i),i \in {\mathbb {N}}$ is a discrete-time Markov chain, with transition probabilities given by

1 $$\begin{aligned} p_{xy} = {\mathbb {P}}(Y(i+1)=y|Y(i)=x) = \frac{q_{xy}}{q_x}, \text{ where } q_x=\sum _{z\in {\mathcal {X}}: z\ne x} q_{xz}. \end{aligned}$$

We now specialise the above general results to the Weighted Voter Model. Let $T_i$ denote the $i^\textrm{th}$ jump time of the Markov process ${\textbf{X}}(t)$, i.e., the $i^\textrm{th}$ time that some agent changes its opinion. Define the discrete-time process $({\textbf{Y}}(i), i\in {\mathbb {N}})$ by setting

$$\begin{aligned} {\textbf{Y}}(0) = {\textbf{X}}(0), \; {\textbf{Y}}(i) = {\textbf{X}}(T_i), i=1,2,\ldots \end{aligned}$$

Then $({\textbf{Y}}(i), i\in {\mathbb {N}})$ is a discrete-time Markov chain whose transition probabilities can be readily calculated from the transition rates of the continuous-time process $({\textbf{X}}(t), t\ge 0)$. Details are in the appendices, for the Weighted Voter Model instantiated on different networks such as complete, regular, and random graphs.

Notice that at each update epoch in the model described above, exactly one agent changes its opinion. This is because the exponential distribution is continuous, and so the probability of two agents updating their opinions at the exact same instant is zero. The above model is called the *asynchronous* discrete-time model (where the discrete time steps are the opinion update epochs). In contrast, in the *synchronous* model, all agents update their opinions simultaneously, in parallel, at each time step. We only consider the asynchronous model in this paper. While the analysis can be extended to the synchronous model, it would detract from the clarity of the exposition; besides, the synchronous model is unrealistic for swarm robotics applications.

With a slight abuse of notation, we shall also refer to the above discrete-time Markov chain as the Weighted Voter Model, it being clear from context whether time is continuous or discrete. In particular, consensus probabilities are the same whether calculated in discrete or continuous time (as we are looking at the exact same process, with the discrete-time version being obtained simply by defining the $i^\textrm{th}$ time step to be the time of the $i^\textrm{th}$ jump in the continuous-time process), and we shall use the discrete-time model for simplicity. When calculating the time to consensus, we shall be interested in actual time rather than number of jumps, as that is usually the performance metric relevant to applications. Hence, we will work with the continuous-time model for calculating time to consensus.

### Consensus probabilities

We now state our main result about consensus probabilities for the model described above.

#### Theorem 1

Consider the Weighted Voter Model on a graph $G=(V,E)$, with rates 1 and $\lambda >1$ associated with opinions *A* and *B*, as described above. Suppose that *G* is a connected, *d*-regular graph for some $d\ge 2$. Consider an initial condition ${\textbf{Y}}(0)$ in which *l* nodes prefer *A* and $N-l$ nodes prefer *B*, where $N=|V|$. Let $T\in {\mathbb {N}}$ denote the random time at which consensus is reached. Then *T* is finite almost surely (a.s.), and the probability of reaching consensus on the worse option, *B*, is given by

2 $$\begin{aligned} {\mathbb {P}}(Y(T)\equiv B):= {\mathbb {P}}(Y_v(T)=B \text{ for } \text{ all } v\in V)= \frac{\lambda ^{N-l}-1}{\lambda ^N-1}, \end{aligned}$$

so that the probability of reaching consensus on the better option is

3 $$\begin{aligned} {\mathbb {P}}(Y(T) \equiv A)= 1-{\mathbb {P}}(Y(T)\equiv B)= \frac{\lambda ^N-\lambda ^{N-l}}{\lambda ^N-1}. \end{aligned}$$

The proof uses martingale-based techniques and is reported in Appendix A. For the benefit of readers who may be unfamiliar with these, we provide a brief, non-technical overview. Loosely speaking, a martingale is a real-valued stochastic process whose expected value satisfies a conservation law, i.e., is constant over time. Intuitively, one may think of a martingale as representing the fortune of a gambler who repeatedly plays a fair game. The gambler may employ different strategies for how much to bet at each time step, but because the game is fair, their expected fortune at the end of a time step is the same as at the beginning. An important theoretical result, known as Doob’s Optional Stopping Theorem, states that not only is the expected value of the martingale the same at any fixed time, but it is also the same at random times satisfying certain conditions, known as stopping times. (The main condition is that the stopping time should not depend on the future of the random process.) In terms of the gambling analogy, it says that a gambler cannot come up with a strategy, no matter how complicated, which delivers a positive expected gain in a fair game. The relevance to our setting is that we can define a function of the number of agents preferring option *B* which is a martingale. By taking the stopping time to be the random time at which consensus is reached, when the number of agents preferring option *B* is either 0 or *N*, depending on whether consensus was reached on option *A* or *B*, the expected value of the martingale at the stopping time is related to the probabilities of reaching consensus on *A* and *B* respectively. By the Optional Stopping Theorem, this is the same as the value of the martingale at time 0, which is just a function of the initial conditions. This enables us to calculate the probability of reaching consensus on each option.

We now comment on some qualitative insights that can be gleaned from Theorem 1.

#### Remarks

1. Taking $l=1$, the theorem says that the probability of reaching consensus on the better option *A* when only a single agent advocates it initially is given by $(\lambda ^{N}-\lambda ^{N-1})/(\lambda ^N-1)$. Notice that this probability is bounded below by
  $$\begin{aligned} \frac{\lambda ^{N}-\lambda ^{N-1}}{\lambda ^N} = \frac{\lambda -1}{\lambda }, \end{aligned}$$
  uniformly in *N*. Thus, the theorem implies that a single agent can persuade an arbitrarily large population of the better choice, with non-vanishing probability.
2. Observe from eqn. (2) that the probability of reaching consensus on *B* is given by
  $$\begin{aligned} \frac{\lambda ^{N-l}-1}{\lambda ^N-1}\le \frac{\lambda ^{N-l}}{\lambda ^N}= \lambda ^{-l}, \end{aligned}$$
  where the inequality holds because $(a-1)/(b-1)\le a/b$ whenever $1<a<b$. Thus, the error probability, of reaching consensus on the worse option, *B*, decays exponentially in *l*, the number of agents initially championing the better option. In particular, if we take $l=\lceil \alpha N \rceil$ for some $\alpha \in (0,1)$, then the probability of reaching consensus on *B* is bounded above by $\lambda ^{-\alpha N}$. In words, if a positive fraction of agents initially prefer the better option, then the error probability, of reaching consensus on the worse option, decays exponentially in the population size. The decay rate only depends on the ratio of site qualities and the initial proportion favouring the better option.

Theorem 1 gives exact results for the best-of-two problem. We now present bounds and approximations for the best-of-*n* problem. Denote the options $1,2,\ldots ,N$, arranged in decreasing order of quality, denoted $q_1>q_2>\ldots >q_N$. (If two or more options have the same qualities, they can be considered as a single option.) We denote the reciprocals of the qualities by $\lambda _i=1/q_i$, $i=1,\ldots ,N$, setting $\lambda _1=1$ w.l.o.g., as in the best-of-two case. Finally, we denote by $N_i>0$ the number of agents preferring option *i*, where $N_1+N_2+\ldots +N_n=N$. We now have the following corollary to Theorem 1.

#### Corollary 1

Consider the Weighted Voter Model algorithm for the best-of-*n* problem on a connected *d*-regular graph with *N* nodes. Suppose that the signalling times for the different options are exponentially distributed with parameters $\lambda _1=1<\lambda _2<\ldots <\lambda _N$. Assume that $N_i>0$ nodes initially favour option *i*, with $N_1+N_2+\ldots +N_k=N$. Then consensus is reached with probability 1. The error probability, of reaching consensus on an option other than 1, the best option, is bounded above by

$$\begin{aligned} {\mathbb {P}}(error) \le \frac{\lambda _2^{N-N_1}-1}{\lambda _2^N-1}. \end{aligned}$$

The corollary says that replacing all preferences for options 3 and worse by preferences for option 2, the second-best option, only makes it harder to converge to the best option. The proof follows a standard coupling argument and is explicated in Appendix A. While the corollary provides a rigorous upper bound on the error probability, this upper bound can be very conservative if options $3,4,\ldots ,n$ are much worse than option 2. This motivates us to propose the following heuristic. Suppose that agents initially preferring options 1 or 2 are frozen in their initial preferences, and continue to signal them, until such time as all other agents have adopted one of these two preferences. This approximates a scenario in which all other options are much worse, and hence have much shorter signalling times. Then, the expected proportion of the remaining agents which adopt preferences 1 and 2 will be exactly their initial proportions, $N_1$ to $N_2$. Thus, we expect to end up with $\frac{N_1}{N_1+N_2}N$ agents having opinion 1 and$\frac{N_2}{N_1+N_2}N$ agents having opinion 2 at the time that all other opinions have disappeared. From this time onward, the process evolves exactly as in the best-of-two setting. Thus, we obtain the following conjecture.

#### Conjecture 1

In the setting of Corollary 1, the error probability, of reaching consensus on an option other than 1, is bounded above by

$$\begin{aligned} {\mathbb {P}}(error) \le \frac{\lambda _2^{\frac{N_2}{N_1+N_2}N}-1}{\lambda _2^N-1}. \end{aligned}$$

### On the time to reach consensus

We now turn to bounding the time to reach consensus, defined as

4 $$\begin{aligned} T=\inf \{t\ge 0: {\textbf{X}}(t) \equiv A \text{ or } {\textbf{X}}(t) \equiv B \}. \end{aligned}$$

Note that we are interested in the actual time taken in the original continuous-time process, though we will make use of the embedded discrete-time jump chain in the analysis. We shall bound ${\mathbb {E}}[T]$, the mean time to reach consensus. As with consensus probabilities, we first consider the complete graph and then move on to regular graphs. In order to analyse arbitrary *d*-regular graphs, we will need the following definition.

**Fig. 1:**
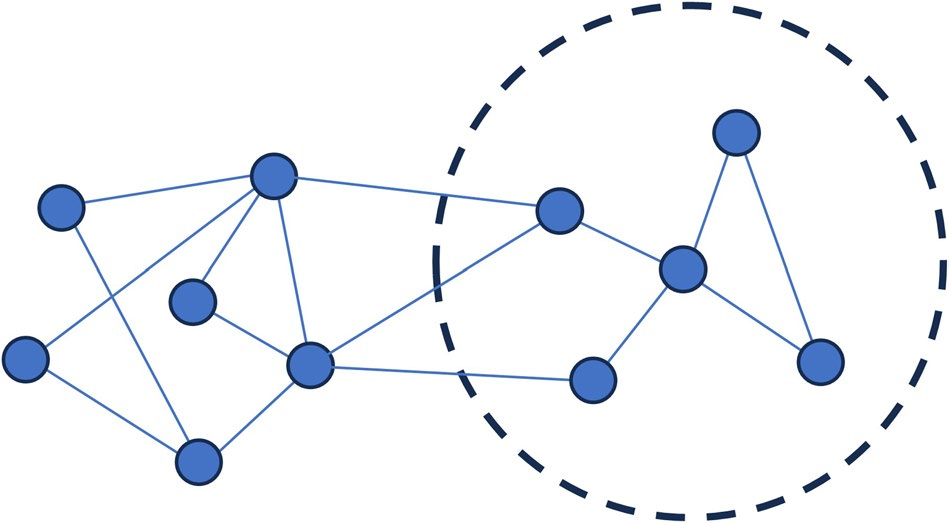
Example of a graph cut with $|S|=5$, $|S^c|=6$ and $|E(S,S^c)|=3$

#### Definition

The isoperimetric constant of a graph $G=(V,E)$ on *N* nodes is defined as

$$\begin{aligned} \eta = \min _{S\subset V:1\le |S| \le N/2} \frac{|E(S,S^c)|}{|S|}, \end{aligned}$$

where $E(S,S^c)=\{ (u,v)\in E: u\in S, v\in S^c \}$ denotes the set of all edges with one endpoint in the subset *S* of the vertex set and the other endpoint in its complement, $S^c$. We use |*S*| to denote the cardinality of a set *S*.

In words, we look at the minimum, over all cuts or bipartitions of the vertex set, of the ratio of the number of edges crossing the cut to the number of vertices in the smaller part. If we think of the number of edges as the perimeter of the set *S*, and the number of vertices as its area, we are seeking the minimum perimeter for a given area. This is known as the isoperimetric problem, whence the constant gets its name. See the example in Fig. 1, which shows a cut with 3 edges crossing it. The two subsets into which the vertex set is divided have cardinality 5 and 6. Hence, for this particular cut, $|E(S,S^c)|/|S|=3/5$, where we take *S* to be the subset consisting of 5 nodes; notice from the definition of the isoperimetric constant that the minimisation is over subsets consisting of no more than *N*/2 nodes. The figure shows just one possible cut. In order to find the isoperimetric constant, we need to consider all possible cuts and determine which one achieves the minimum.

#### Theorem 2

Consider a Weighted Voter Model on a connected, undirected graph $G=(V,E)$ with *N* nodes, and rates 1 and $\lambda >1$ associated with options *A* and *B*, as above. Let the consensus time *T* be defined as in eqn. (4). Then, if *G* is the complete graph on *N* nodes, we have

$$\begin{aligned} {\mathbb {E}}[T] \le \frac{2(1+\log N)}{\lambda -1}. \end{aligned}$$

If *G* is a *d*-regular graph with isoperimetric constant $\eta >0$, then

$$\begin{aligned} {\mathbb {E}}[T] \le \frac{2d(1+\log N)}{\eta (\lambda -1)}. \end{aligned}$$

The proof is in Appendix B.

#### Remarks

1. The first claim of the theorem says that, on a complete graph, the time to reach consensus in the Weighted Voter Model grows only logarithmically with the population size. This is in stark contrast to the classical voter model, where it grows linearly (Liggett, 1985).
2. The second claim of the theorem says that consensus also happens in logarithmic time on graph sequences whose isoperimetric constant is bounded away from zero, uniformly in *N*. Such graph families are known as expanders. Examples include the complete graph and random *d*-regular graphs for $d\ge 3$ (Diestel, 2005). Counterexamples include the ring and the *d*-dimensional torus, which is 2*d*-regular. By torus, we mean a hypercube within the *d*-dimensional lattice, with opposite faces identified.
3. The isoperimetric constant of the complete graph is $\lceil N/2 \rceil$, with the minimum being attained by any subset comprised of $\lfloor N/2 \rfloor$ nodes. Thus, the general bound in the second claim is loose by a factor of 2 for the complete graph.
4. While the theorem provides bounds on the time to consensus, it is straightforward to numerically compute the exact mean time to consensus on the complete graph, starting from an arbitrary initial state. Letting *A*(*t*) denote the number of agents preferring site *A* at time *t*, we see that *A*(*t*) is a Markov process on $\{ 0,1,\ldots ,N \}$, with absorbing states at 0 and *N*, which are reachable from all other states. Let *P* denote the transition probability matrix of the embedded discrete-time chain restricted to the transient states $\{ 1,\ldots ,N-1 \}$. Then, the number of visits to state *N* before absorption, for the chain started in state *j*, is given by the $jk^\textrm{th}$ element of the matrix $(I-P)^{-1} = \sum _{t=0}^{\infty } P^t$. The expected time to absorption starting in state *j* is obtained by summing over all states *k* the expected number of visits to *k* times the mean time spent in state *k* on each visit. The latter, the mean residence time, is given by eqn. (17) in Appendix B.

### Robustness of consensus probabilities and times

The two theorems in the previous subsections give an exact expression for the probability of reaching consensus on either option, and an upper bound on the time to reaching consensus, for the Weighted Voter Model on a complete graph or on a *d*-regular graph. The theorems were stated and proved for static graphs, which do not change over time. The robots in a swarm typically move, and so their neighbourhoods change over time. A careful look at the proofs will show that they still hold provided that the graphs at all time instants satisfy the conditions of the theorem, i.e., that they are all *d*-regular and have isoperimetric constant equal to, or larger than, $\eta$. In fact, the theorems continue to hold even if an adversary chooses the graphs, provided that the adversary has to satisfy these constraints.^2^ In addition, of course, it is assumed that the adversary does not know the future, e.g., which node will be the next to update its state. More formally, any strategy adopted by the adversary must be adapted to the filtration ${\mathcal {F}}_{t-}=\sigma ({\textbf{X}}(s),s<t)$, where $\sigma ({\textbf{X}}(s),s<t)$ denotes the sigma-algebra generated by the process up to, but not including, the time instant *t*.

The restriction of the results to regular graphs is limiting as it is unlikely that the neighbourhoods generated by randomly moving robots are always of the exact same size. We now show that the results are robust to small deviations from regularity; they can be extended to graphs which are “approximately regular” in the sense that $d_{\max }/d_{\min }$, the ratio of the maximum to minimum node degree, is not much larger than 1. In particular, if it is smaller than $\lambda$, the ratio in quality of the two sites (and hence of the mean signalling time of the two options), then the same results hold qualitatively; the probability of reaching consensus on the worse option decays exponentially in population size, and the time to reach consensus grows logarithmically. We make this precise below.

#### Theorem 3

Consider a Weighted Voter Model on a connected, undirected graph $G=(V,E)$, with options *A* and *B* being signalled for *Exp*(1) and $Exp(\lambda )$ random times respectively, with $\lambda >1$. Suppose that

$$\begin{aligned} \mu := \frac{\lambda d_{\min }}{d_{\max }} > 1. \end{aligned}$$

Let *T* denote the random time to reach consensus. Then, *T* is finite a.s. and, conditional on *k* nodes initially preferring option *A*, we have

$$\begin{aligned} {\mathbb {P}}({\textbf{X}}(T)\equiv B) \le \frac{\mu ^{N-k}-1}{\mu ^N-1}, \quad {\mathbb {E}}[T] \le \frac{2d_{\max }(\mu +1)(1+\log N)}{\eta (\mu -1)(\lambda +1)}, \end{aligned}$$

where $\eta$ denotes the isoperimetric constant of *G*.

The proof is in Appendix C.

#### Remark

If $d_{\max }=d_{\min }=d$, then the graph is *d*-regular, $\mu =\lambda$, and we recover the results in Theorems 1 and 2.

## Consensus with noisy measurements

We have assumed so far that the quality of a site can be captured by a single numerical value, and that this value is assessed perfectly by each agent. This assumption is unrealistic, both for biological and robotic systems. In the real world, we expect that site quality measurements are imperfect and noisy. Our motivation in this section is to relax the assumption that quality measurements are perfect by allowing for random measurement errors.

We now set out our precise assumptions about the nature of measurement errors. We shall retain the assumption that site quality can be represented by a single number. While this is questionable as multiple criteria enter into any assessment, and these might be weighted differently by different agents, we nevertheless retain it for simplicity. Moreover, the role played by quality assessments is to determine the length of time for which an agent signals a specific option before updating its opinion. Thus, any method chosen by an agent to determine this time could be interpreted as implying a numerical judgement of site quality. Next, we assume that distinct measurements of the same site are identically distributed, irrespective of which agent makes them. Moreover, distinct measurements, whether by the same or different agents, are mutually independent. Notice that we do not allow for agent heterogeneity. One agent may not be consistently prone to over- or under-estimating site quality relative to another agent. One agent may not consistently differ from another in terms of giving greater preference to one of the sites. In other words, all variability in assessments of options quality is purely random and not a reflection of agent heterogeneity.

We now make our assumptions mathematically precise. We assume that measurements of site *A* yield estimates which are independent and identically distributed (i.i.d.) non-negative random variables denoted $T^A_1, T^A_2,\ldots$. We let ${\hat{F}}_A(\cdot )$ denote their (cumulative) distribution function (cdf). If an agent obtains an estimate $T^A$, then it signals a preference for $T^A$ for an $Exp(1/T^A)$ random time before updating its preference. Similarly, measurements of site *B* yield i.i.d. estimates $T^B_1, T^B_2,\ldots$, with distribution function ${\hat{F}}_B(\cdot )$. The notation is chosen to reflect the fact that these random variables represent the mean length of time for which preferences for *A* or *B* are maintained, and thus have the physical dimension of time. Define the random variables $R^A_i=1/T^A_i$ and $R^B_i=1/T^B_i$; *R* represents rates. We denote the distribution functions of $R^A$ and $R^B$ by $F_A$ and $F_B$ respectively. Finally, we assume throughout this section that agents are the nodes of a complete graph, i.e., any two agents can communicate directly. Extending the analysis to general graphs is an open problem.

If agents live on the complete graph and site quality measurements are perfect, then the number of agents with either opinion, say *A*, evolves as a Markov process. This is no longer the case if measurements are noisy. In order to have the Markov property, the state space needs to be augmented to keep track of the random variables *T* or *R* corresponding to the measurements by each node. The augmented state space is too large for direct analysis, and does not appear to yield any convenient martingales which would enable an exact analysis. Therefore, we focus on heuristics for large populations.

Consider a ‘large’ population of *N* agents, a ‘small’ number *k* of whom initially prefer the ‘better’ option, *A*; we define what we mean by better in Conjecture 2 below. More precisely, we are interested in a limiting regime in which *k* is fixed, while *N* tends to infinity. While our theoretical analysis is conducted in this large population limit, we present simulations employing moderate and realistic numbers of agents. The simulation results show good agreement with the theoretical predictions.

The main quantity of interest is the probability of reaching consensus on *A*. Following the terminology for the generalised Moran process, which is closely related to the Weighted Voter Model studied in this paper, we will call this the “fixation probability”; in the Moran process, which was outlined in the introduction, it is the probability that a fitter mutant takes over a population.

Let *A*(*t*) and *B*(*t*) denote the number of agents with preference *A* and *B* respectively, at time *t*. For a node $v$, let $R_v(t)$ denote the rate at which it stops signalling and updates its opinion. Suppose that only a small number, $A(t)=j$, of nodes prefer option *A*. Fix one such node, $v$. Now, the total rate at which some node *w* with opinion *B* at time *t* updates its opinion by copying $v$ is given by $\sum _{w\in B(t)} R_w(t)/(N-1)$, as the updating node has probability $1/(N-1)$ of choosing to copy node $v$. It may appear at first glance that $(R_w(t), w\in B(t))$ are i.i.d. with distribution $F_B$, but this is incorrect. When an agent *w* adopts opinion *B*, it samples a rate $R_w$ from the distribution $F_B$; if $R_w=x$, then it retains this value for an *Exp*(*x*) random time, with mean 1/*x*. Thus, in a population of such agents at equilibrium, smaller values of *x* are more likely to be observed. To be precise, in equilibrium, an agent *w* sampled uniformly at random from agents with opinion *B*, will have rate parameter $R_w$ distributed according to the *size-biased distribution* $G_B$, given by

5 $$\begin{aligned} dG_B(x) = \frac{1}{x}dF_B(x) \Bigm / \int _0^{\infty } \frac{1}{y}dF_B(y). \end{aligned}$$

To see why the size-biased distribution is given by the above expression, note that an agent with preference *B* samples a quality ‘close to’ *x* with probability $dF_B(x)$ and signals *B* for a random time with mean 1/*x*. This explains why $dF_B(x)$ is multiplied by 1/*x* in the numerator to give the proportion of *B* agents whose sampled quality is close to *x* and which are still signalling that value. The denominator is a normalising constant, needed to ensure that $\int _0^{\infty } dG_B(x)=1$, i.e., that $G_B$ is a probability distribution.

The total rate $\lambda$ at which agents with opinion *B* contact a specific agent $v$ is given by

6 $$\begin{aligned} \begin{aligned} \lambda&= \frac{1}{N-1}\sum _{w\in B(t)} R_w(t) \\&\approx \frac{1}{N-j}\sum _{w\in B(t)} R_w(t)\approx {\mathbb {E}}[R_w] \\&= \int _0^{\infty } x dG_B(x) = \frac{ \int _0^{\infty } dF_B(x)}{\int _0^{\infty } \frac{1}{y}dF_B(y)} \\&= \frac{1}{{\mathbb {E}}[1/R_B]} = \frac{1}{{\mathbb {E}}[T_B]}, \end{aligned} \end{aligned}$$

where the first approximation in the second line holds because the number of agents, *N*, is assumed to be large; the second approximation in this line follows by the law of large numbers as the number of terms in the sum is $N-j$, the number of nodes holding opinion *B*.

We call each such node *w* which copies v a ‘child’ of $v$. When it copies $v$, it also assesses the quality of site *A*, and acquires a rate $R_w$ which specifies how long it will signal *A* until updating its opinion. We call the numerical value $R_w$, which is a random sample from the distribution $F_A$, the ‘type’ of node *w*. At the end of an $Exp(R_w)$ random time period, node *w* will stop signalling and update its opinion again by copying a randomly chosen node. As the vast majority of nodes are in state *B*, it is very likely to switch state to *B*. We ignore the small probability $j/(N-1)$ that it remains in state *A*, and say that it ‘dies’ at this time.

It should be clear from the description above that, subject to the approximations made therein, the number of nodes, $(A(t), t\in {\mathbb {R}}_+)$, with opinion *A* evolves as a multitype branching process.^3^ An individual of type *r* lives for an *Exp*(*r*) random time. During this time, it gives birth to new individuals according to a Poisson process of intensity $\lambda$, given by eqn.(6). Each child has a random type drawn from the distribution $F_A$, independent of the type of its parent. This branching process yields a good approximation to *A*(*t*) as long as *A*(*t*) remains small. The approximation becomes poorer as *A*(*t*) becomes larger. We are interested in whether *A*(*t*) first reaches 0 (consensus is reached on *B*) or *N* (consensus is reached on *A*). If *A* is the better option, then it is very unlikely to reach consensus on *B* once *A*(*t*) has become moderately large. Based on this intuition, we shall approximate the probability of reaching consensus on *B* by the extinction probability of the multitype branching process described above. We make this precise in the following conjecture.

### Conjecture 2

Consider the noisy Weighted Voter Model comprised of *N* agents on the complete graph, $K_N$. When an agent adopts opinion *A* (resp. *B*), it samples a random variable $T_A$ (resp. $T_B$) from distribution ${\hat{F}}_A$ (resp. ${\hat{F}}_B$) and signals the chosen opinion for the corresponding length of time. It then adopts the opinion of a randomly chosen individual from the population. Suppose ${\mathbb {E}}[T_A]>{\mathbb {E}}[T_B]$; we then say that *A* is the *better* option. Let $\alpha _k(N)$ denote the probability of reaching consensus on option *A* (fixation) if started with *k* individuals with opinion *A* and $N-k$ with opinion *B*, with initial values of $T_A$ and $T_B$ sampled as above. Let $\pi$ denote the extinction probability in a multitype branching process in which each individual has offspring at rate $\lambda$ given by eq. (6), the offspring type is sampled from $F_A$ and a type *r* individual lives for a random time with *Exp*(*r*) distribution. Then, for any fixed $k\in {\mathbb {N}}$

$$\begin{aligned} \lim _{N\rightarrow \infty } \alpha _k(N) = 1-\pi ^k. \end{aligned}$$

The conjecture motivates us to study the associated branching process. We shall make use of the following general result about extinction probabilities in multitype branching processes, stated in terms of generating functions. Generating functions play an important role in the analysis of branching processes and the calculation of extinction probabilities. Let *X* be an integer-valued random variable with probability mass function $p_X$, i.e., $p_X(j)={\mathbb {P}}(X=j)$, $j\in {\mathbb {Z}}$. The generating function of *X*, denoted $G_X$, say, is defined by $G_X(u)={\mathbb {E}}[u^X] = \sum _{j=-\infty }^{\infty } p_X(j)u^j$; the domain is the set of *u* for which the sum is absolutely convergent. Notice that this is the same as the *z*-transform of $p_X$, the mass function. Generating functions are a powerful analytical tool because the generating function of the sum of independent random variables is the product of their generating functions.

### Theorem 4

(Harris (1963), Theorem 7.1) Consider a multitype branching process with *J* types. Let $\varvec{\xi }_i= (\xi _{i1},\ldots ,\xi _{iJ})$ denote a random vector with the joint distribution of the number of offspring of types $1,\ldots ,J$ of a single type *i* individual. Suppose the branching process is positively regular, i.e., there is a $t\in {\mathbb {N}}$ such that, for any *i* and *j*, there is a non-zero probability that a type *i* individual has a type *j* descendant in generation *t*.

Define the generating functions

$$\begin{aligned} G_i({\textbf{u}}) = {\mathbb {E}}\Bigl [ u_1^{\xi _{i1}} u_2^{\xi _{i2}} \cdots u_j^{\xi _{ij}} \Bigr ], \text{ where } {\textbf{u}}= (u_1,\ldots ,u_J) \in [0,1]^J. \end{aligned}$$

Then, the fixed point equations $G_i({\textbf{u}}) = u_i$, $i=1,\ldots ,J$, have a solution $\varvec{\pi }\in [0,1]^J$ such that, for any other solution ${\textbf{u}}^* \in [0,1]^J$, we have $\pi _i\le u^*_i$ for all $i=1,\ldots ,J$. Moreover, $\pi _i$ is the probability of extinction starting with a single type *i* individual.

In the noisy Weighted Voter Model, we potentially have a continuum of types rather than just a finite number. Nevertheless, we extend the above theorem to our setting and establish the following result.

### Theorem 5

Consider the multitype branching process described in Conjecture 2. Let $R_A$ denote a random variable with the type distribution, $F_A$. Assume that $\lambda {\mathbb {E}}[1/R_A]>1$ (i.e., *A* is the better site, in the language of the Weighted Voter Model). Then, the fixed point equation

7 $$\begin{aligned} c = {\mathbb {E}}\Bigl [ \frac{\lambda c}{\lambda c+R_A} \Bigr ], \end{aligned}$$

has a unique positive solution $c^*$, and the extinction probability of the branching process, started with a single type *r* individual, is given by $\pi _r = \frac{r}{r+\lambda c^*}$. Consequently, if the branching process starts with a single individual whose type is sampled from $F_A$, then the extinction probability is given by

$$\begin{aligned} \pi = {\mathbb {E}}\Bigl [ \frac{R_A}{R_A+\lambda c^*} \Bigr ] = 1-c^*. \end{aligned}$$

### Proof

We first show existence and uniqueness of solutions to the fixed point equation. Define $g(c) = {\mathbb {E}}\bigl [ \frac{\lambda }{\lambda c+R_A} \bigr ]$, and notice that $g:(0,\infty )\rightarrow (0,\infty )$ is a strictly decreasing and convex (hence, continuous) function. Furthermore, *g*(*c*) tends to zero as *c* tends to infinity, whereas, as *c* tends to zero, *g*(*c*) tends to $\lambda {\mathbb {E}}[1/R_A]$, which is strictly bigger than 1 by the assumption of the theorem. Hence, $g(c)=1$ has a solution by the continuity of *g*, which is unique by the strict monotonicity of *g*. But *c* solves (7) if and only if $g(c)=1$.

It remains to show that the relation between the extinction probability and the solution $c^*>0$ of (7) is as claimed in the theorem. We first suppose that the random variable $R_A$ is discrete and can only take values in a finite set. Consider a single individual of type *r*, i.e., with an *Exp*(*r*) lifetime. As noted above, it has children according to a Poisson process of intensity $\lambda$. Denote the number of children of a type *r* individual by $\xi _r$. Then, $\xi _r+1$ has a $Geom(\frac{\lambda }{\lambda +r})$ distribution, i.e., a geometric distribution with parameter $\frac{r}{\lambda +r}$ and mean $\frac{\lambda +r}{r}$. One way to see this is to note that, irrespective of how many children a type *r* individual has already had, it has at least one more before it dies if an $Exp(\lambda )$ random variable representing the time to the next birth is smaller than an independent *Exp*(*r*) random variable denoting its residual lifetime. The probability of this event is $\frac{\lambda }{\lambda +r}$, by well-known properties of exponential random variables. If we call the complementary event, of death before having one more child, a success, then the number of children is one less than the number of independent Bernoulli trials, with success probability $\frac{r}{\lambda +r}$, required to obtain the first success. Finally, each child has a type drawn from the distribution $F_A$, independent of the type of the parent.

Next, we compute the extinction probability of the branching process model described above, assuming that the distribution $F_A$ is supported on finitely many points, denoted $0\le r_1<\ldots <r_J$, with respective probabilities $p_1,\ldots ,p_J$. Then, as noted above, the number of children of a type *i* individual has a shifted $Geom(\frac{r_i}{\lambda +r_i})$ distribution. As the types of the children are chosen independently, the number of children of each type has a multinomial distribution conditional on the total number. Using this, we can compute the generating functions:

8 $$\begin{aligned} \begin{aligned}&G_i({\textbf{u}}) = \sum _{N=0}^{\infty } \frac{r_i}{\lambda +r_i} \Bigl ( \frac{\lambda }{\lambda +r_i} \Bigr )^N \sum _{\begin{array}{c} {N_1,\ldots ,N_J:}\\ {N_1+\ldots +N_J=N} \end{array}} \frac{N!}{N_1!\cdots N_J!} (p_1 u_1)^{N_1}\cdots (p_J u_J)^{N_J} \\&\quad = \sum _{N=0}^{\infty } \frac{r_i}{\lambda +r_i} \Bigl ( \frac{\lambda }{\lambda +r_i} \Bigr )^N (p_1 u_1+\ldots +p_J u_J)^N \\&\quad = \frac{r_i}{\lambda +r_i} \Bigl ( 1-\frac{\lambda \sum _{j=1}^J p_j u_j}{\lambda +r_i} \Bigr )^{-1} = \frac{r_i}{r_i+\lambda (1-\sum _{j=1}^J p_j u_j)}. \end{aligned} \end{aligned}$$

We now invoke Theorem 4. The assumption of positive regularity holds with $t=1$, since each individual has positive probability of having children of any type. Hence, $\pi _i$, the probability of extinction starting with a single individual of type *i*, can be obtained by solving the fixed point equations

9 $$\begin{aligned} \pi _i = \frac{r_i}{r_i+\lambda c}, \text{ where } c:= 1-\sum _{j=1}^J p_j\pi _j. \end{aligned}$$

It follows that *c* must solve the fixed point equation

$$\begin{aligned} \begin{aligned} c&= 1-\sum _{j=1}^J p_j \frac{r_j}{r_j+\lambda c} = \sum _{j=1}^J p_j \Bigl (1-\frac{r_j}{r_j+\lambda c} \Bigr ) \\&= \sum _{j=1}^J p_j \frac{\lambda c}{r_j+\lambda c} = {\mathbb {E}}\Bigl [ \frac{\lambda c}{\lambda c+R_A} \Bigr ]. \end{aligned} \end{aligned}$$

This establishes the claim of the theorem when $R_A$ takes only finitely many values.

Our goal is to extend the result to the general case, when $R_A$ takes a continuum of values. Our approach is to approximate a general $R_A$ by a random variable that only takes finitely many values. More precisely, we consider a sequence of better and better approximations, as follows. We first bound $R_A$ by some number *M* (i.e., consider the random variable, $\min \{R_A,M\}$), and approximate it by a random variable taking values in the finite set, $\{ 0,\epsilon ,2\epsilon ,\ldots ,M \}$. We get one such approximation for each choice of $\epsilon$ and *M*. By considering a sequence of such choices with $\epsilon$ tending to zero and *M* to infinity, we approximate $R_A$ to ever greater precision. The details are fleshed out below. We emphasise that $\epsilon$ and *M* are purely artifacts used in the proof and not related to any parameters in the swarm system.

We use monotonicity of the extinction probability in the stochastic order, as detailed below, to extend the result to the setting where $R_A$ takes values in a bounded interval, $[0,R_{\max }]$. Let ${\tilde{R}}_A$ be a non-negative random variable. We say that the distribution of ${\tilde{R}}_A$ stochastically dominates that of $R_A$, denoted $R_A\preceq {\tilde{R}}_A$, if ${\mathbb {E}}[g(R_A)]\le {\mathbb {E}}[g(\tilde{R}_A)]$ for every non-decreasing function *g* for which the expectations are defined; see, e.g, Müller and Stoyan (2002) for properties of stochastic orders. In addition to the definition, we shall use the following property in our proof: if $R_A\preceq \tilde{R}_A$, then we can couple them (define them on the same probability space) in such a way that $R_A \le {\tilde{R}}_A$ almost surely (a.s.). We now compare multitype branching processes where the offspring types have distribution $R_A$ with those where they have distribution ${\tilde{R}}_A$. Since $T_A=1/R_A \ge 1/{\tilde{R}}_A$, individuals in the ${\tilde{R}}_A$ process have shorter lifetimes, and hence fewer offspring, a.s. It follows that the extinction probabilities, ${\tilde{\pi }}_r$, associated with a type *r* individual in the ${\tilde{R}}_A$ process satisfy ${\tilde{\pi }}_r \ge \pi _r$ whenever $R_A \preceq {\tilde{R}}_A$.

We can approximate the bounded random variable $R_A$ from below and above (a.s.) by random variables $R^-_{\epsilon }$ and $R^+_{\epsilon }$ taking values in finite subsets of $\{ 0,\epsilon , 2\epsilon , \ldots \}$; we can further choose them in such a way that they converge in probability to $R_A$ as $\epsilon$ tends to zero. Let $c^-_{\epsilon }$ and $c^+_{\epsilon }$ denote the solutions of eqn. (7) with $R_A$ replaced by $R^-_{\epsilon }$ and $R^+_{\epsilon }$ respectively. Since $R^-_{\epsilon } \le R_A$, we have

$$\begin{aligned} 1 = {\mathbb {E}}\Bigl [ \frac{\lambda }{\lambda c^-_{\epsilon } + R^-_{\epsilon }} \Bigr ] \ge {\mathbb {E}}\Bigl [ \frac{\lambda }{\lambda c^-_{\epsilon } + R^A} \Bigr ] =g(c^-_{\epsilon }). \end{aligned}$$

As noted above, *g* is a decreasing function and $c^*$ is the unique positive solution of $g(c^*)=1$. Hence, we have $c^*\le c^-_{\epsilon }$. Similarly, we get $c^*\ge c^+_{\epsilon }$.

Thus, we have sandwiched the extinction probability in the branching process with lifetimes $1/R_A$ between those with lifetimes $1/R^-_{\epsilon }$ and $1/R^+_{\epsilon }$; we have similarly sandwiched the solutions of the corresponding fixed point equations. Now, $R^-_{\epsilon }$ and $R^+_{\epsilon }$ are supported on finitely many points, and so their extinction probabilities are related to the solution of the fixed point equations as stated in the theorem. In order to extend this to $R_A$, all that remains is to show that $c^-_{\epsilon }$ tends to $c^+_{\epsilon }$ as $\epsilon$ tends to zero. But this is straightforward from the continuity of *g*, noted earlier. This completes the proof of the theorem for distributions $R_A$ with bounded support.

It only remains to extend the proof to distributions with unbounded support. Clearly, $\min (R_A, M)$ is an increasing function of $M\ge 0$, and increases to $R_A$ as *M* tends to infinity. Hence, extinction probabilities with the corresponding lifetimes are also an increasing function of *M*. Let $c^M$ solve (7) with $R_A$ replaced by $\min (R_A,M)$; as before, $c^*$ denotes the solution for $R_A$. We have proved that $c^M$ describes extinction probabilities when the reciprocal of the lifetime is given by the bounded random variable $\min (R_A,M)$. It is easy to see that $c^M$ decreases to $c^*$ as *M* increases to infinity. This completes the proof for unbounded distributions. $\square$

Denote the *n*-fold iterate of the function *g* by $g^{(n)}$, i.e., $g^{(1)}=g$, and $g^{(n)}=g\circ g^{(n-1)}$. Suppose that the condition ${\mathbb {E}}[1/R_A]>1/\lambda$ is satisfied. Then, it can be seen from the monotonicity of *g* that $g^{(n)}(0)$ increases monotonically to $c^*$, while $g^{(n)}(1)$ decreases monotonically to $c^*$. Thus, iterating *g* from the initial conditions 0 and 1 yields a numerical procedure which is guaranteed to converge to $c^*$ while also bracketing it, and hence providing error bounds, at each iteration.

### Impact of noise on consensus probability

Our motivation in this section was to quantify the impact of measurement noise on consensus in the Weighted Voter Model, as it is unrealistic to assume that agents, biological or robotic, can assess site quality perfectly, without error. Conjecture 2 related the consensus probability to the extinction probability in a related branching process, which in turn was obtained in Theorem 5 via the solution of a fixed-point equation. Assuming the conjecture is true, the theorem shows that the probability of reaching consensus on the worse site decays exponentially in the number of agents who initially prefer the better site. This result is consistent with findings from agent-based simulations (Valentini et al., 2014), which show that the Weighted Voter Model is robust to measurement noise, at least when initialized with equal numbers of agents preferring each site. Nevertheless, an interesting qualitative question is whether measurement noise decreases the probability of reaching consensus on the better site, and whether more noise decreases it more, as one might expect. In order to address this, we need to make precise what we mean by “more noise”.

Let *X* and *Y* be random variables with distributions $F_X$ and $F_Y$. We say that *X* (resp. $F_X$) is dominated by *Y* (resp. $F_Y$) in the convex stochastic order, written $X {\preceq }_{cx}Y$, if ${\mathbb {E}}[g(X)] \le {\mathbb {E}}[g(Y)]$ for all convex functions *g* for which the expectations exist (Müller & Stoyan, 2002). We say that a distribution ${{\hat{F}}}^2_A$ for site quality measurements is noisier than a distribution ${{\hat{F}}}^1_A$ if ${{\hat{F}}}_A^1 {\preceq }_{cx}{{\hat{F}}}_A^2$. Recall that site qualities correspond to the length of time for which an agent signals that site, and is the reciprocal of the rates used in Theorem 5 above.

The following result formalizes the intuition that noisier measurements make it harder to reach consensus on the better site.

#### Theorem 6

Consider two scenarios for the Weighted Voter Model, with common distribution ${{\hat{F}}}_B$ for measurements, $T_B$, of the quality of the worse site *B* but different distributions ${{\hat{F}}}_A^1$ and ${{\hat{F}}}_A^2$ for $T_A^1$ and $T_A^2$, denoting measurements of the quality of the better site, *A*. Let $\lambda =1/{\mathbb {E}}[T_B]$ as in (6).

For $i=1,2$, let $\pi ^i$ denote the extinction probability in the corresponding branching process, when started with a single individual of random type sampled from ${{\hat{F}}}_A^i$. If ${{\hat{F}}}_A^1 {\preceq }_{cx}{{\hat{F}}}_A^2$, then $\pi ^1 \le \pi ^2$.

The proof is in Appendix D. The theorem says that, if measurements are noisier, then the extinction probability of the branching process is greater; if Conjecture 2 is true, then this implies that the probability of reaching consensus on the better site is smaller.

## Simulations

In this section, we present simulations of the Weighted Voter Model on different graph topologies, in both noise-free and noisy settings, in order to validate the analytical results from the previous sections. We plot consensus probabilities and times as a function of various model parameters, and compare the simulation results with theoretical predictions and bounds from the preceding sections. Each point plotted in the figures below represents the average of 1000 trials. The code for all the simulations is available at https://github.com/emmavalla/Weighted_Voter_Model.git.

As in previous sections, we will assume that the respective site qualities are $q_A =1 \ge q_B > 0$. We let *N* denote the total number of agents. In each simulation, *k* agents^4^ are initialized with a preference for the higher quality site *A*, and $N-k$ agents with a preference for the lower quality site, *B*.

The *N* agents are simulated using two arrays. The first array keeps track of each agent’s preferred site, while the second keeps track of its current estimate of the quality of that site. These site quality estimates parametrise the probability distributions of the agents’ signalling times. An adjacency matrix describes the network of communication links between agents. The simulation proceeds in discrete time steps or rounds. In each round, a single agent is chosen to update its preference. The choice of agent is weighted according to their site quality estimates; the probability that agent *i* will be chosen is inversely proportional to $q_i$, agent *i*’s current estimate of the quality of its current preferred site. In simulations with no noise, agent *i*’s new quality estimate is equal to the true quality of its preferred site. In simulations with noise, this estimate is sampled from the appropriate distribution.

### Complete graph with noise-free measurements

**Fig. 2:**
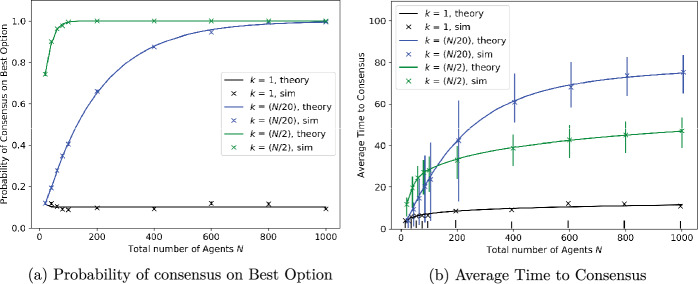
Consensus probability and time versus total number of agents, *N*, for different numbers of agents, *k*, initially preferring better option. Units for time are scaled so that mean signalling time is 1 for the better option and 0.9 for the worse option

**Fig. 3:**
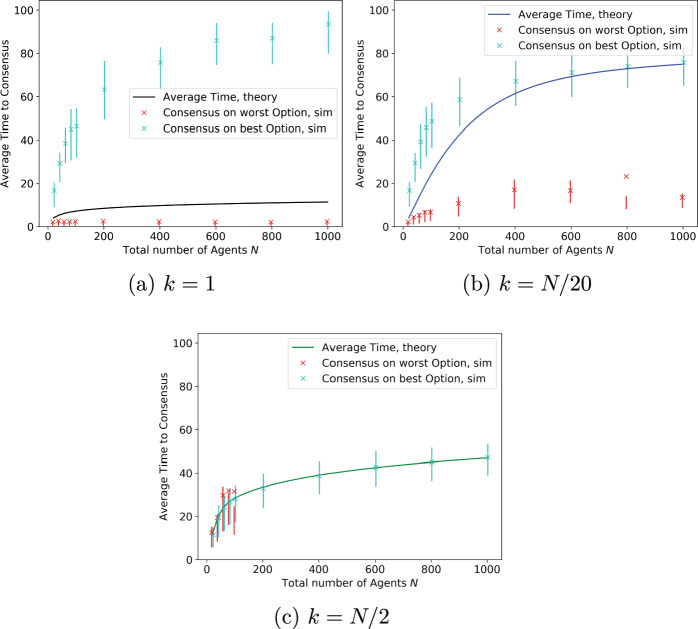
Average time to consensus as in Fig. 2b, split between the cases when consensus is reached on the worst option or the best option

**Fig. 4:**
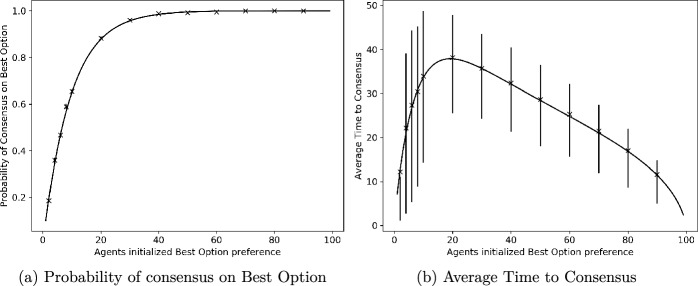
Consensus probability and time versus total number of agents, *k*, initially preferring better option. Total number of agents is $N=100$. Units for time are scaled so that mean signalling time is 1 for better option and 0.9 for worse option

**Fig. 5:**
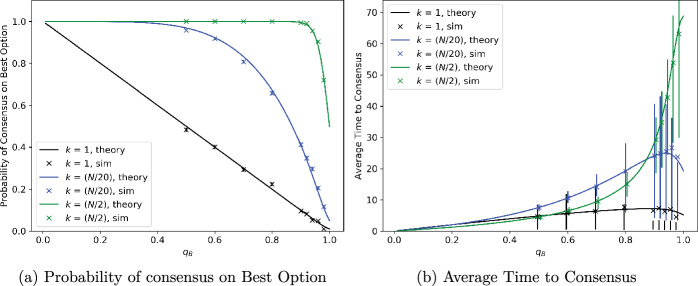
Consensus probability and time versus quality (mean signalling time, $q_B$) for worse site, for different numbers, *k*, of agents initially preferring better site. Mean signalling time of better site, $q_A$, is normalised to 1. Total number of agents is $N=100$. In the case $k=1$ in the right subplot, the mean lies outside the interquartile range for a few values. See Remark 1 for an explanation

We simulated the Weighted Voter Model on complete graphs with a varying number of nodes, and for site qualities measured without noise. We took $\lambda =1.111$, i.e., the quality of the worse site is 90% that of the better site. The results of the simulations are plotted in Figs. 2, 3, 4 and 5, alongside theoretical results derived from Theorems 1, 2.

Figure 2 shows the performance of the algorithm as the number of agents, *N*, ranges from 10 to 1000. We plot the probability of reaching consensus on the best site, i.e., the proportion of simulation runs in which all agents reach consensus on the best site, in Fig. 2a and the average time to reach consensus in Fig. 2b, as a function of the number of agents. The three plots in each figure pertain to different numbers of agents, *k*, being initialised with a preference for the best site; from bottom to top, these are $k = 1$, $k = N/20$, and $k = N/2$. The crosses come from simulations, while the solid line is the analytical result from Theorem 1. Note that when *k* scales as a proportion of *N*, as with $k = N/2$ and $k = N/20$, the probability of reaching consensus on the better site approaches 1 as *N* grows to infinity; if *k* is fixed and only *N* grows, then this probability approaches a fixed non-zero value which depends on *k* and is strictly smaller than 1. In all cases, theory closely matches the simulations. Moreover, the theoretical calculations are almost instantaneous, while the simulations are time-consuming as the number of agents becomes large. As the variable being simulated in Fig. 2a is categorical (consensus is either on the best option or not) rather than numerical, there are no error bars associated with the simulations. Now, the number of simulations converging to the best option is a binomial random variable with parameters 1000 (the number of simulations) and *p*, the unknown probability of reaching consensus on the best option; it has mean 1000*p* and variance $1000p(1-p)$. Hence, the *proportion* of simulations reaching consensus on the best option is well approximated by a normal random variable with mean ${\hat{p}}$ and variance ${\hat{p}}(1-{\hat{p}})/1000$, where ${\hat{p}}$ is the estimate of *p* plotted in the figure. The implied width of the inter-quartile range, $1.35\sqrt{{\hat{p}}(1-{\hat{p}})/1000}$, is too small to be visible in the figure and is hence not plotted.

Figure 2b shows how the average time to consensus grows with the number of agents. The crosses represent the mean from simulations and the bars the interquartile range, namely the range from the first to third quartile, while the solid lines are numerical results calculated as described in the remarks following Theorem 2. The mean time to consensus is not monotonic in the initial condition. The figure shows that, for larger values of *n*, simulations with $k = N/20$ take a longer time to reach consensus than those with either $k=1$ or $k = N/2$. The reason for this becomes clear from Fig. 3, which separately depicts the average time to consensus on the better or worse site for various values of *k*. These show that, when *k* is small, consensus is reached quickly on the worse option but slowly on the better one. As *k* is increased, the time to reach consensus on the better option decreases, but this is initially offset by the increasing probability of reaching consensus on the better option. Once *k* becomes sufficiently large, the time needed to reach consensus on the better option dominates, and decreases in *k*.

This also explains why, in the case where $k=1$ in Fig. 2b, the cross depicting the mean time to reach consensus does not lie within the error bar depicting the interquartile range. Agents are reaching consensus on the best option with a probability of less than 0.25, but this happens much more slowly and, therefore, greatly skews the overall mean time. However, once *k* is large enough, this event becomes highly unlikely and the average is dominated by the time to reach consensus on the better site, which decreases as *k* (the number of agents initially preferring the better site) increases. Finally, the reader may notice that even in the plots for time to consensus on the worse option in Fig. 3, the cross depicting the mean value is offset significantly from the centre of the corresponding error bar, and sometimes lies outside the error bar! This is again a reflection of high variability in the random time to reach consensus, which substantially affects the mean but not the interquartile range. We chose to depict the mean rather than the median in the plots because our theoretical analysis pertains to the mean. As this phenomenon is observed repeatedly in many of our plots, we summarise the explanation in a remark below for easy reference.

#### Remark 1

In many plots, the cross depicting the mean value is offset significantly from the centre of the corresponding error bar, and sometimes lies outside the error bar! This is a reflection of high variability in the random quantity being plotted, which affects the mean much more than the interquartile range. In the case of consensus times, this usually occurs for parameter values for which the time to reach consensus on the better option is large, but the probability of doing so is small.

Figure 4 shows the performance of the algorithm for different numbers of agents, *k*, initialised with a preference for the better site; *k* runs from 1 through 99, with the total number of agents, *N*, being fixed at 100. Figure 4a shows that the probability of reaching consensus on the better site quickly approaches 1 as the value of *k* increases. Figure 4b gives the average time to consensus, with the bars depicting the interquartile range. As remarked above, the mean time to consensus is not monotonic in *k* but reaches a peak around $k=20$. Again, we note the good match between theoretical predictions and results from simulations for both consensus probabilities and times.

Figure 5 shows the performance of the algorithm over a range of relative site qualities. The quality of the worse site varies from 0 to 1, the total number of agents *N* is 100, and simulations are performed for $k = 1, N/20, N/2$ agents initially preferring the better site, *A*. Figure 5a shows the probability of reaching consensus on the better site, *A*. When the quality of site *B* is 1, i.e., both sites are equally good, the probability of reaching consensus on site *A* is equal to *k*/*N*. As the quality of site *B* decreases, the probability of reaching consensus on site *A* increases to 1, rapidly for $k=N/2$, gradually for $k = N/20$ and very slowly for $k=1$. The theoretical curves in this figure come from Theorem 1, and closely match the simulation results.

Figure 5b depicts the mean and interquartile range of the time to consensus. This mostly increases as $q_B$, the quality of the worse site increases, but exhibits a small dip as $q_B$ approaches 1. It is intuitive that consensus should take longer as the difference in site quality decreases; mathematically, the drift towards the better site becomes weaker. The dip in the time to consensus when $q_B$ is close to 1, and $k=1$ or *N*/20, is due to a sharp increase in the probability of reaching consensus on site *B*, which is only slightly worse as $q_B$ gets close to 1. This does not affect the $k = N/2$ case as reaching consensus on *B* is not faster from this initial condition.

### Non-instantaneous measurements

In the results presented above, we ignored the time required to measure site quality as the analysis in this paper was carried out under the assumption that site quality could be measured instantaneously. This assumption is relaxed in the simulation results presented below, in which there is a lag between the time that a node or agent updates its preference and the time that it starts signalling that preference. The lag represents the time required to measure site quality, and is assumed to be exponentially distributed, with the same parameter for both options. Moreover, lags are mutually independent across agents and measurements.

**Fig. 6:**
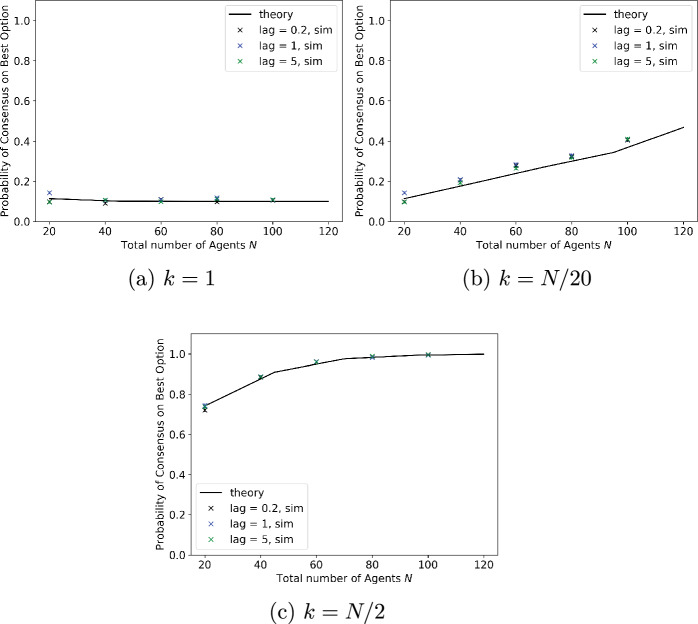
Effect of lags between agent updating and signalling opinions on the probability of reaching the best option. Number of agents is $N=100$, of whom *k* initially prefer better site. Mean signalling time is 1 for better site, 0.9 for worse site

**Fig. 7:**
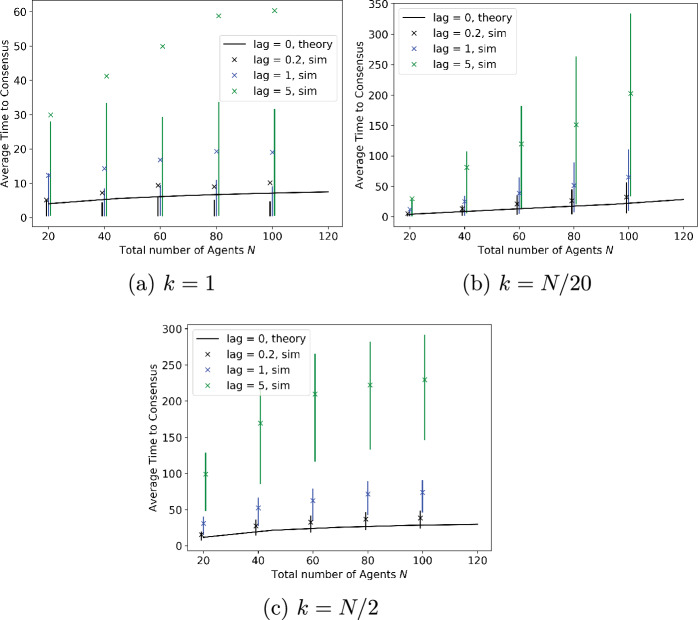
Effect of lags between agent updating and signalling opinions on the time to consensus. Number of agents is $N=100$, of whom *k* initially prefer better site. Mean signalling time is 1 for better site, 0.9 for worse site. In some cases when lag=0, the cross depicting the mean lies outside the error bar. See Remark 1 for an explanation

The probability of reaching consensus on the better option is plotted in Fig. 6, and the time to reach consensus in Fig. 7, as the number of agents is varied. All results are based on 1000 simulation runs. The signalling time is taken to be exponentially distributed with mean 1 for the better option and 0.9 for the worse option. Lags are also exponentially distributed; results are plotted for three different mean lags, of 0.2, 1 and 5, representing measurement times which are respectively small relative to the signalling time, comparable to it, and significantly larger. While we expect the first of these to be the most common scenario in practice, the simulations explore how robust the algorithm is to the time required for measurement. The three subplots in each figure correspond to different initial conditions, where the number of agents initially preferring the best option is 1, 5% of all agents, or half of all agents. Figure 6 shows that the time required to measure site quality does not affect the probability of reaching consensus on the better option, while Fig. 7 shows that, unsurprisingly, it significantly increases the time needed to reach consensus. A theoretical analysis of consensus time for this model is an open problem for future research.

### Multiple opinions: best-of-*n* problem

**Fig. 8:**
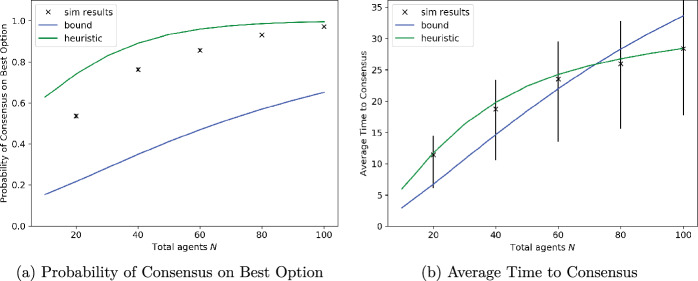
Effects of multiple opinions on the complete graph. *N*/10 agents initially prefer each of the 10 sites, with mean signalling times 0.1, 0.2, ..., 1

We now revert to the setting of instantaneous measurements but consider the general best-of-*n* problem instead of the best-of-two problem that we have mainly studied in this paper. Figure 8 shows simulation results for the best-of-*n* algorithm with $n=10$. The options have qualities $q_{10} = 0.1, q_9 = 0.2,\ldots ,q_{1} = 1$, while the total number of agents is varied as $N=20,40,\ldots ,100$. The system is simulated starting with an equal number of agents initially favour each of the ten options.

The proportion of 1000 simulations in which consensus was reached on the best option, 1, is depicted in Fig. 8a, along with the theoretical lower bound on this probability from Corollary 1 and the heuristic approximation to it from Conjecture 1. While the lower bound does indeed lie below the values from simulations, it is rather conservative as argued in Sect. 2, whereas the heuristic provides a good approximation, especially in larger systems. The time to reach consensus is depicted in Fig. 8b, along with two heuristics obtained as follows. Inspired by Corollary 1, one of them applies Theorem 2 with an initial condition in which all agents whose initial preference is not for the best option are initiated with the second-best option, option 2. The other heuristic follows Conjecture 1 in reassigning nodes preferring options 3–10 equally to one of the two best options, 1 or 2. The results show that the former heuristic provides a better approximation to the consensus time; the latter ignores the time needed to go from the actual initial condition to the vicinity of the approximating one in which all but the two best options have disappeared from the system.

### Regular graphs with noise-free measurements

**Fig. 9:**
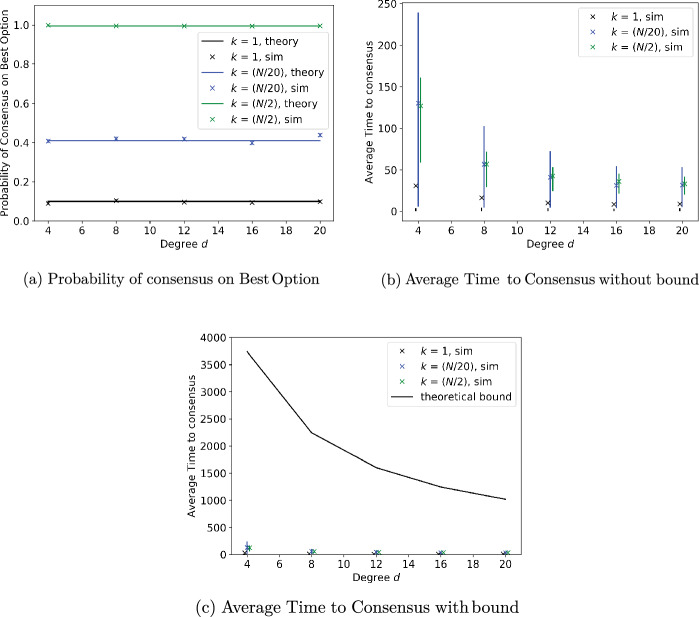
Consensus on expanded cycle graph with $N=100$ agents, versus *d*, number of nearest neighbours to which each node is connected; *k* agents initially prefer better site. Time units normalised so that mean signalling time is 1 for the better site, 0.9 for the worse. See Remark 1 for an explanation of why the mean consensus time for $k=1$ in the middle subplot lies outside its error bars

We simulated the Weighted Voter Model on an expanded cycle graph with $N=100$ nodes, which consists of nodes $0,1,\ldots ,N-1$, arranged in a ring, with each node *i* having edges to its *d* nearest neighbours in the ring, namely nodes $i - \frac{d}{2},\dots ,i-1,i+1,\dots ,i + \frac{d}{2}$, modulo *N*. The graph is *d*-regular as each node has *d* neighbours; its isoperimetric constant can be calculated exactly and is equal to $\frac{2}{N}{\sum _{i=1}^{d/2} 2i }$. In Fig. 9, we plot the probability of reaching consensus on the better option and the time to do so, alongside the theoretical bounds from Theorem 1 and 2. We see that the simulated consensus probabilities in Fig. 9a closely match the theoretical bounds, whereas the simulated consensus times in Fig. 9b are an order of magnitude smaller. This suggests that the theoretical bound on consensus times is conservative, and that the Weighted Voter Model algorithm can reach consensus far more rapidly in practice than the theoretical guarantees.

**Fig. 10:**
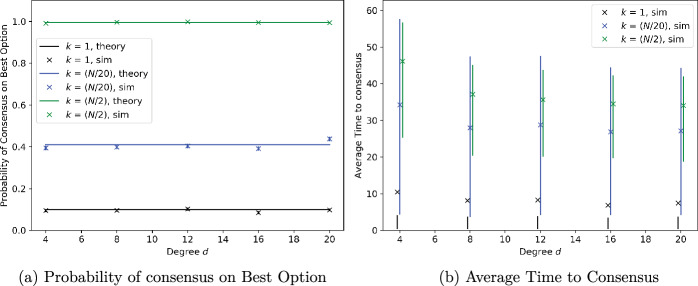
Consensus probabilities and times for $N = 100$ agents forming a random regular graph, and different numbers, *k*, initially preferring better option. Time units chosen so that mean signalling time is 1 unit for better option, 0.9 for worse option. See Remark 1 for an explanation of why the mean consensus time for $k=1$ lies outside its error bars

Figure 10 depicts simulations carried out on *d*-regular random graphs, namely those in which all nodes have the same degree, *d*. The number of agents is fixed at $N =100$, and simulations are carried out for $d = 4, 8, 12, 16, 20$. In each trial, a new random regular graph is generated with the desired degree *d*, on which the Weighted Voter Model then evolves. Each simulation point is therefore the average of these single trajectories on 1000 such random graphs. Consensus probabilities in Figure  10a are plotted alongside the theoretical predictions from Theorem 1, which they match closely. They show that the probability of reaching consensus on the best option does not vary with *d* but is equal to that for the complete graph, shown in Figure  2a.

Figure  10b shows the average time to consensus, which is seen to decrease with the degree, *d*. We have not plotted the theoretical bound in this figure as it requires knowledge of the isoperimetric constant, whose computation is known to be NP-hard (Garey et al., 1976). Nevertheless, it has been shown in (Bollobás, 1988, Corollary 2) that with high probability (namely, with probability tending to 1 as *N* tends to infinity), the isoperimetric constant of the random *d*-regular graph on *N* nodes is bounded below by $d/2-\sqrt{d\log 2}$. Substituting this in Theorem 2, the mean time to consensus is bounded with high probability by $4(1+\log N)/(\lambda -1)(1-\sqrt{\frac{\log 2}{d}})$. Evaluating this for the parameter values in our simulations yields a consensus time bound which decreases from 346 when $d=4$ to 250 when $d=20$. These bounds are again conservative when compared to the simulation results in Fig. 10b, albeit not as much as for the expanded cycle graph.

**Fig. 11:**
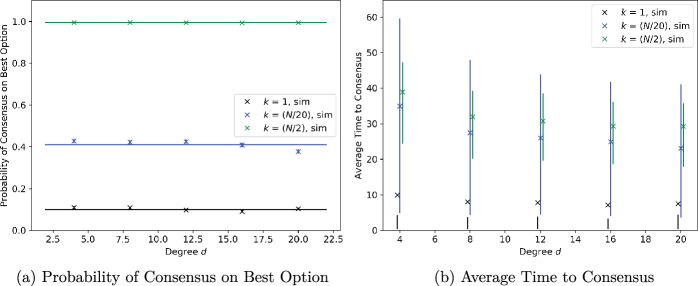
Effect of node degree on consensus probabilities and times in dynamic random regular graphs, in which edges are partially re-sampled at every jump. Number of agents is $N=100$, of whom *k* initially prefer better site. Mean signalling time is 1 for better site, 0.9 for worse site. See Remark 1 for an explanation of why the means for $k=1$ lie outside their error bars

### Dynamic networks

The simulations reported in Figs. 9 and 10 were carried out on static networks and do not capture changes in network topology caused by robots moving to measure site quality. In order to study these effects, we perform simulations on a random regular graph, with partial edge re-sampling at every opinion update. Whenever an agent updates its opinion, it severs communication links with its *d* neighbours and chooses *d* new neighbours uniformly at random. The rest of the network is then re-wired to preserve regularity, as follows. The *d* neighbours with which an updating node *u* severed connection are matched with its *d* new neighbours. Suppose a node *v* with which *u* severed connection is matched with node *w* with which *u* formed a new connection. Then, an edge is removed between *w* and one of its neighbours, *x*, chosen at random, and a new edge is created between *x* and *v*. This ensures that all node degrees are unchanged after the rewiring.

We simulated the Weighted Voter Model on a network evolving as described above to model robot movement. The results are shown in Fig. 11. Observe from Fig. 11a that the probability of consensus on the best option matches theoretical predictions for static regular graphs. Next, comparing Fig. 11b for the evolving graph to Fig. 10b for static random regular graphs, we see that the time to reach consensus is shortened. These results suggest that the theoretical analysis for static networks yields predictions that are accurate (for consensus probabilities) or conservative (for consensus times) for dynamic networks. In particular, theoretical predictions continue to be useful as performance guarantees in the more realistic setting of networks evolving to model robot movement.

### Non-regular graphs with noise-free measurements

The objective of this section is to explore the performance of the Weighted Voter Model algorithm on communication graphs which are only approximately regular. Our main finding from simulations is that the probability of reaching consensus on the better option is almost the same as in regular graphs, which provides empirical evidence for the robustness of the Weighted Voter Model algorithm on a variety of graph topologies. The time to consensus remains short for a wide range of parameter values, but does grow sharply when the number of edges becomes very small, i.e., when the graphs become nearly disconnected.

**Fig. 12:**
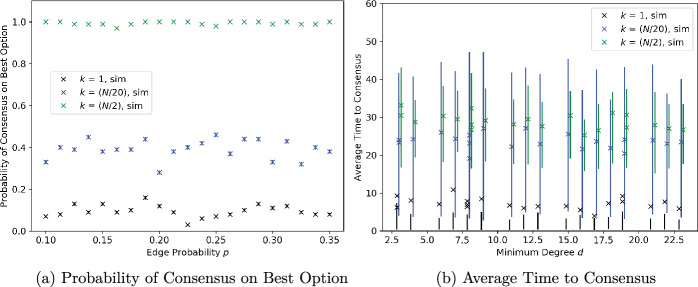
Effect of node degree on consensus probabilities and times in Erdős-Rényi Random Graphs. Number of agents is $N=100$, of whom *k* initially prefer better site. Mean signalling time is 1 for better site, 0.9 for worse site. See Remark 1 for an explanation of why the means for $k=1$ lie outside their error bars

**Fig. 13:**
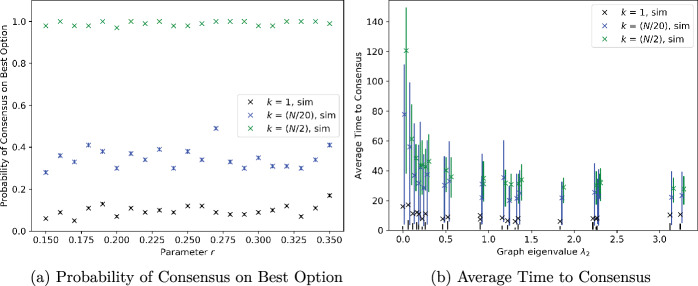
Effect of node degree on consensus probabilities and times in Random Geomteric Graphs. Number of agents is $N=100$, of whom *k* initially prefer better site. Mean signalling time is 1 for better site, 0.9 for worse site. See Remark 1 for an explanation of why the means for $k=1$ lie outside their error bars

We report on simulations of the Weighted Voter Model on Erdős-Rényi (ER) random graphs in Fig. 12, and on random geometric graphs in Fig. 13, both with noiseless site quality measurements. An ER random graph on *N* nodes, with parameter $p \in [0,1]$, is defined as the graph obtained by putting an edge between any pair of nodes with probability *p*, independent of all other edges. A random geometric graph on *N* nodes, with parameter $r>0$, consists of *N* nodes placed independently and uniformly at random on the unit square; any two nodes separated by a distance smaller than *r* are joined by an edge, while nodes further apart than *r* have no edge between them. The simulations are performed for $k = 1$ and $k = 5$ nodes initialised with a preference for the better site, *A*.

For the ER graph simulations, we chose uniformly spaced values of *p* in the range $0.1-0.4$. We generated one ER graph for each value of *p* and ran 1000 iterations of the Weighted Voter Model on it; the average time to reach consensus and the probability of reaching consensus on site *A* were calculated from these iterations. Analogous simulations were performed for random geometric graphs, for values of *r* ranging over $0.15-0.35$.

The results for ER graphs are plotted in Fig. 12 and for random geometric graphs in Fig. 13. Figures 12a and 13a show that the probability of reaching consensus on the better site depends on *k*, the number of agents initially preferring the better site, but is fairly insensitive to the parameters *p* and *r* of the random graphs; it is also very similar to that for random regular graphs, depicted in Fig. 10. Thus, the simulation results reinforce the message of Theorem 3, that the probability of reaching consensus on the better site is fairly insensitive to the network topology but only depends on the difference in site qualities and on the initial number of agents preferring the better site.

In Figs. 12b and 13b, we plot the average time to consensus against the minimum node degree for ER graphs, and against the second smallest eigenvalue of the Laplacian for random geometric graphs. We chose these as crude proxies for the isoperimetric constant, which is used in Theorem 2 to bound the time to consensus; we did not attempt to compute the isoperimetric constant as it is known to be NP-complete for general graphs (Garey et al., 1976). The figures show little dependence of the consensus time on the chosen proxies over a wide parameter range. However, Fig. 13b does show a sharp increase in the consensus time as the second eigenvalue of the Laplacian becomes close to zero, which corresponds to a nearly disconnected network.

### Complete graph with Gamma-distributed noise

**Fig. 14:**
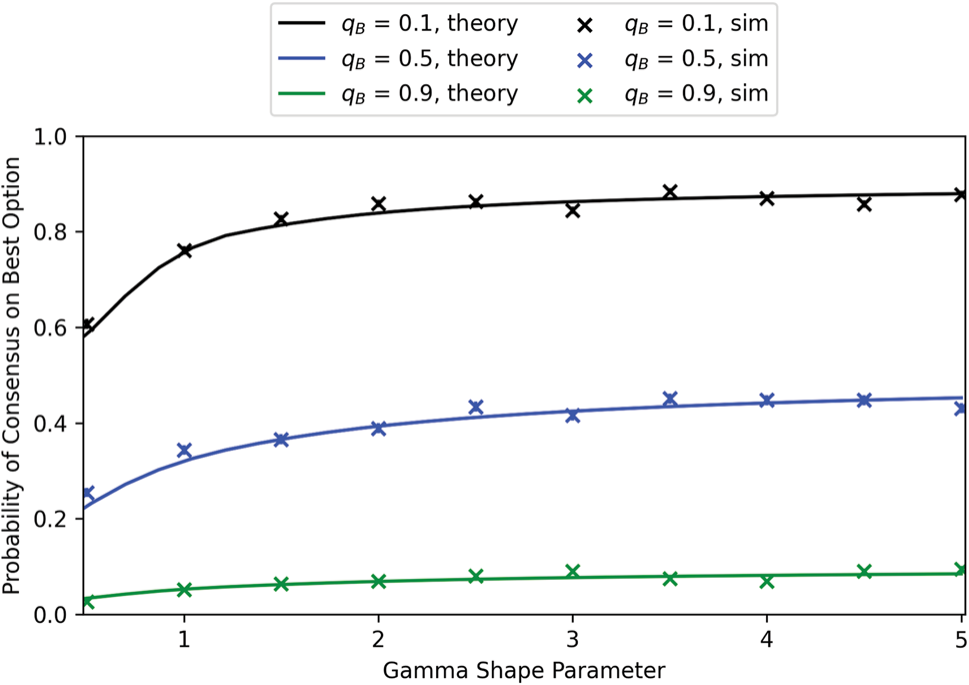
Consensus probabilities for $N=100$ agents on a complete graph, with varying measurement noise. Site quality measurements yield random results with a gamma distribution, with means $q_A=1$ and $q_B=0.1, 0.5, 0.9$. Smaller values of the shape parameter correspond to more noisy measurements

The simulation results presented so far involve agents making perfect assessments of site quality. This in unrealistic, and unlikely to be realised in practice. This motivates us, in this section, to present results of simulations performed on the complete graph, with noisy measurements of site quality as described in Sect. 3. In the set-ups considered previously, the initial condition was fully described by the number of agents preferring each site. Now, we additionally need to specify the initial site quality measurement of each agent, or equivalently, the residual length of time for which they will hold the initial preference before changing state. It would be natural to consider the steady state performance of the algorithm, and hence to sample the random initial site quality measurements from their steady state distribution. An important subtlety is that this is not the same as sampling from the site quality distributions, ${\hat{F}}_A$ and ${\hat{F}}_B$. Indeed, as noted in Sect. 3, higher values of site quality will be held by agents for longer; hence, steady state site quality values will have the size-biased distribution

$$\begin{aligned} d{\hat{G}}_B(t) = \frac{td{\hat{F}}_B(t)}{\int _0^{\infty } sd{\hat{F}}_B(s)ds} = \frac{td{\hat{F}}_B(t)}{{\mathbb {E}}[T_B]}, \end{aligned}$$

and similarly for ${\hat{G}}_A$. The equation above is the analogue, for holding times, of eqn. (5), which pertained to rates. We use the above size-biased distribution to simulate the initial site quality estimates.

The results are presented in Fig. 14, for site quality measurements sampled from gamma distributions with varying shape parameters, and with scale parameters chosen to keep the mean value of the distribution equal to the true site quality. We simulated scenarios in which the mean values of the quality of site *B* was equal to 0.1, 0.5, and 0.9. With only one agent initially preferring the better site, $k = 1$. The theoretical results depicted in the figure are derived using the branching process approximation, i.e., Conjecture 2 and Theorem 5. The simulations results are very close to the theoretical predictions and provide strong evidence in support of the conjecture. The results also show that the probability of reaching consensus on the better option is fairly robust to noise, and only decreases for rather small values of the shape parameter, which correspond to rather large measurement noise.

## Conclusions

We studied a biologically inspired algorithm, known as the Weighted Voter Model, for the best-of-*n* problem in collective decision-making. This algorithm has received much attention in swarm robotics. Evaluations of its performance to date have relied on either agent-based simulations, analysis of differential equations arising in the large-population limit under well-mixing assumptions, or numerical evaluation of Markov chain models. Simulations and numerical evaluation are computationally expensive, whereas the differential equations approach is hard to justify for small to moderate numbers of agents, and fails to account for limited communication range between agents. The main contribution of this work is to present a rigorous mathematical analysis of this algorithm under a variety of scenarios.

We explicitly model locality of agent interactions by representing agents as nodes of a network and the ability of two agents to communicate directly by an edge between them. Thus, the network captures communication constraints, bringing it closer to real-world systems where physical space impacts which robots can communicate with each other. We first consider the complete graph, in which all pairs of agents can communicate directly. This corresponds to a well-mixed dynamical system such as assumed in most existing analyses of the Weighted Voter Model. We also initially restrict ourselves to the case where site qualities are measured perfectly, without error or noise. For agents located on a complete graph and making noise-free measurements of site quality, we present exact results for the probability of consensus on the best site, and tight bounds on the time to consensus.

We then extend the analysis to regular graphs, again obtaining exact results for the probability of reaching consensus on the best site. We also obtain upper bounds on the time to consensus, but simulations show that these can be very conservative. Regular networks correspond to the assumption that every agent communicates with exactly the same number of other agents. This is clearly unrealistic in practice, though we can expect that in many situations there will not be huge variability in the number of agents with whom each agent communicates. With this motivation, we consider networks which are only approximately regular, and derive bounds on consensus probabilities and times in this setting.

Next, we return to the complete graph setting but relax the assumption of noise-free measurements, as it is unrealistic to assume that agents can evaluate site qualities perfectly in practical applications. We present an analysis of consensus probabilities in the noisy setting using a branching process approximation. Simulations suggest that it is close to exact on systems of moderate size. We also show that, as expected, greater measurement noise reduces the probability of reaching consensus on the better site; in order to do so, we first make precise the comparison of different noise distributions by proposing the use of the convex stochastic order.

Our theoretical analysis yields results on the performance of the Weighted Voter Model which can be computed quickly, without the need for time-consuming numerical computations or agent-based simulations. By considering a variety of graph models and incorporating measurement noise, we make our analysis relevant to a range of real-world scenarios. An important qualitative insight arising from our analysis is the robustness of the Weighted Voter Model to the graph topology and to measurement errors.

Our results are restricted to static networks. Realistically, in robot swarms (or insect colonies), the set of agents with which each agent interacts will evolve over time as the agent moves. Moreover, some of these agents may be under the control of a malicious adversary. Hence, an interesting direction for future work is to study dynamic networks, including ones over which an adversary can exert some degree of control. We briefly mentioned in Sect. 2.3 that our analysis could be extended to such settings, but doing so in detail is an interesting problem for future research.

We have only studied the simplest version of the Weighted Voter Model, in which agents are directly recruited from one belief to another (or possibly the same) by interaction with a single other agent. There has been work on more intricate models in which agents may spontaneously change opinion, and in which there is cross-inhibition as well as recruitment (Reina et al., 2017; Talamali et al., 2021); the latter work intriguingly suggests that constraints on communication can improve consensus. Hence, a second avenue for future research is to analyse more complex variants of voter models, as well as to understand the impact of communication constraints in detail. Exact analysis of models with cross-inhibition is challenging; see (Perron et al., 2009) for the model with two opinions. This fact motivated interest in majority voter models, which exhibit similar convergence probabilities (Cruise & Ganesh, 2014) and are easier to analyse. Majority voter models create a drift towards the majority opinion in the Markov chain dynamics. If the majority happens to favour a sub-optimal option, this competes with the drift towards the better option and gives rise to a phase transition, leading to very slow convergence, exponentially long in the population size, in certain parameter regimes (Mukhopadhyay, 2023). Thus, a challenge for future research is to extend the analyses to models with cross-inhibition, and to design rules that ensure rapid convergence.

Finally, it remains an open problem to provide a rigorous justification for the branching process approximation to consensus probabilities in the noisy setting.

## Data Availability

N/A.

## Appendix A: Consensus probabilities on regular graphs

### Proof of Theorem 1

Let $B(t) = |\{ v\in V: {\textbf{Y}}_v(t)=B \}|$ denote the number of nodes with preference *B* at the start of the $t^\textrm{th}$ epoch in the discrete-time jump chain, ${\textbf{Y}}(t)$. We start by proving that the process $(M(t), t\in {\mathbb {N}})$, defined by $M(t)=\lambda ^{B(t)}$ is a martingale, i.e.,

10 $$\begin{aligned} {\mathbb {E}}[M(t+1)|{\textbf{Y}}(s), s\le t] = M(t) \; \forall \; t \in {\mathbb {N}}. \end{aligned}$$

We will first show this when *G* is a complete graph.

Let ${\textbf{Y}}(t)$ be a configuration in which *m* nodes have opinion *B* and $N-m$ have opinion *A*. In continuous time, each node with opinion *A* updates its opinion at rate 1, copying the opinion of a *B* node with probability $\frac{m}{N-1}$ and of an *A* node with the residual probability. It is only in the former event that its opinion changes. Thus, the total rate at which some one of the $N-m$ nodes with opinion *A* changes opinion to *B* is $m(N-m)/(N-1)$. Similarly, each of the *m* nodes with opinion *B* updates opinion at rate $\lambda$, copying an *A* node and changing opinion to *A* with probability $(N-m)/(N-1)$, while copying a *B* node and retaining its opinion with the residual probability. Hence, the total rate at which some *B* node changes opinion to *A* is $m(N-m)\lambda /(N-1)$. Putting these together, the total rate at which *B*(*t*) increases from *m* to $m+1$ is $m(N-m)/(N-1)$, while the total rate at which it decreases from *m* to $m-1$ is $m(N-m)\lambda /(N-1)$. In the discrete-time Markov chain obtained by embedding at the jump times of the continuous-time chain, when some node changes opinion, the transition probabilities are proportional to these transition rates. Thus, we get

11 $$\begin{aligned} \begin{aligned} {\mathbb {P}}(B(t+1)=m+1|B(t)=m)&=\frac{\frac{m(N-m)}{N-1}}{\frac{m(N-m)\lambda }{N-1}+\frac{m(N-m)}{N-1}} = \frac{1}{\lambda +1},\\ {\mathbb {P}}(B(t+1)=m-1|B(t)=m)&= \frac{\frac{m(N-m)\lambda }{N-1}}{\frac{m(N-m)\lambda }{N-1}+\frac{m(N-m)}{N-1}} = \frac{\lambda }{\lambda +1}. \end{aligned} \end{aligned}$$

Substituting in $M(t)=\lambda ^{B(t)}$ and using the Markov property, we get

$$\begin{aligned}&{\mathbb {E}}[M(t+1)-M(t)|{\textbf{Y}}(s), s\le t, B(t)=m] = {\mathbb {E}}[M(t+1)-M(t)|{\textbf{Y}}(t), B(t)=m)] \\&\quad =\frac{1}{\lambda +1}(\lambda ^{m+1}-\lambda ^m) + \frac{\lambda }{\lambda +1}(\lambda ^{m-1}-\lambda ^{m}) = 0, \end{aligned}$$

where we have obtained the second line by multiplying the possible values of the random variable $M(t+1)-M(t)=\lambda ^{B(t+1)}-\lambda ^{B(t)}$ by their corresponding probabilities from eqn. (11). As the above holds for all ${\textbf{Y}}(t)$, and corresponding $m\in \{ 0,1,\ldots ,N \}$, it follows that *M*(*t*) is a martingale if the graph *G* is complete.

The proof when *G* is a regular graph is similar. As before, consider a configuration ${\textbf{Y}}(t)$ with $B(t)=m$. Denote by $E(t)=E({\textbf{Y}}(t))$ the number of edges whose endpoints have different opinions in the configuration ${\textbf{Y}}(t)$, and by $E_v(t)$, $v\in V$, the number of neighbours of $v$ whose opinion differs from $v$. Then,

12 $$\begin{aligned} \sum _{v:Y_v(t)=A} E_v(t)= \sum _{v:Y_v(t)=B} E_v(t) = E(t), \end{aligned}$$

since each edge with differing endpoints gets counted once in each sum.

Now, each node $v$ with opinion *A* updates its opinion at rate 1, choosing to copy a *B* node with probability $E_v(t)/d$ as it has $E_v(t)$ edges to *B* nodes and *d* edges in total and picks a neighbour at random. With the residual probability, it copies another *A* node and retains its opinion. Thus, the total rate at which some *A* node changes opinion to *B* is given by

13 $$\begin{aligned} \sum _{v:Y_v(t)=A} 1\cdot \frac{E_v(t)}{d} = \frac{E(t)}{d}, \end{aligned}$$

where the equality follows from eqn. (12). Similarly, each *B* node $v$ updates opinion at rate $\lambda$, copying an *A* node and changing opinion with probability $E_v(t)/d$, and retaining its opinion with the residual probability. Hence, the total rate at which some *B* node changes opinion to *A* is given by

14 $$\begin{aligned} \sum _{v:Y_v(t)=B} \lambda \cdot \frac{E_v(t)}{d} = \frac{\lambda E(t)}{d}. \end{aligned}$$

By embedding at the jump times of this continuous-time Markov chain, we obtain a discrete-time chain whose transition probabilities are proportional to the above transition rates. Thus, we obtain from eqns. (13) and (14) that

15 $$\begin{aligned} \begin{aligned} {\mathbb {P}}(B(t+1)=m+1|{\textbf{Y}}(t),B(t)=m)&= \frac{E(t)/d}{(E(t)/d)+(\lambda E(t)/d)} = \frac{1}{1+\lambda }, \\ {\mathbb {P}}(B(t+1)=m-1|{\textbf{Y}}(t),B(t)=m)&= \frac{\lambda E(t)/d}{(E(t)/d)+(\lambda E(t)/d)} = \frac{\lambda }{1+\lambda }. \end{aligned} \end{aligned}$$

Notice that the transition probabilities in eqn. (15) for the regular graph are exactly the same as in eqn. (11) for the complete graph. Thus, by a similar calculation to earlier,

$$\begin{aligned}&{\mathbb {E}}[M(t+1)-M(t)|{\textbf{Y}}(s), s\le t, B(t)=m, E(t)] = {\mathbb {E}}[M(t+1)-M(t)|{\textbf{Y}}(t), B(t)=m, E(t)] \\&\quad = \frac{1}{1+\lambda }(\lambda ^{m+1}-\lambda ^m) + \frac{\lambda }{1+\lambda }(\lambda ^{m-1}-\lambda ^m) = 0. \end{aligned}$$

This completes the proof that $M(t)=\lambda ^{B(t)}$ is a martingale on any regular graph.

Next, define $T=\inf \{ t\in {\mathbb {N}}: {\textbf{Y}}(t)\equiv A \text{ or } {\textbf{Y}}(t) \equiv B \}$ to be the first time that all nodes have the same opinion, which is an absorbing time for this Markov chain. Then *T* is a stopping time, i.e., the occurrence or otherwise of the event $\{ T\le t\}$ is determined by $\{ {\textbf{Y}}(s), s\le t \}$. Moreover, *T* is finite with probability 1; in fact, ${\mathbb {E}}[T]$ is finite since the expected amount of time spent in any transient state is finite, and there are only finitely many such states (see, e.g., Norris (1997)). Finally, $M(\cdot )$ is a bounded martingale, as it can only take values between 1 and $\lambda ^N$. Hence, the conditions for Doob’s Optional Stopping Theorem are satisfied, and we obtain that ${\mathbb {E}}[M(T)]=M(0)=\lambda ^{B(0)}$. If $B(0)=N-k$, then,

$$\begin{aligned} \lambda ^{N-k} = {\mathbb {E}}[M(T)] = 1\cdot {\mathbb {P}}({\textbf{Y}}(T)\equiv A) + \lambda ^N {\mathbb {P}}( {\textbf{Y}}(T)\equiv B), \end{aligned}$$

and so,

$$\begin{aligned} {\mathbb {P}}({\textbf{Y}}(T)\equiv B) = \frac{\lambda ^{N-k}-1}{\lambda ^N-1}. \end{aligned}$$

This completes the proof of the theorem. $\square$

### Proof of Corollary 1

Let ${\textbf{X}}(t)$, $t\in {\mathbb {R}}_+$, denote the continuous-time Markov chain describing the evolution of the best-of-*n* system, which we call the original system. We shall construct a best-of-two system $\tilde{{\textbf{X}}}$, termed the coupled system, on the same probability space as ${\textbf{X}}$ in such a way that the following property holds for all $t\ge 0$:

16 $$\begin{aligned} \tilde{X}_v(t)=1 \; \Rightarrow \; X_v(t)=1 \quad \forall \; v\in V. \end{aligned}$$

In words, if any node $v$ prefers option 1 at time *t* in the coupled system, $\tilde{{\textbf{X}}}$, then it also does so in ${\textbf{X}}$, the original system. Consequently, if consensus is reached on option 1 in the coupled system, it is also reached on option 1 in the original system. It follows from this that the probability of reaching consensus on option 1 is at least as large in the original system as in the coupled system, which is what is claimed in the corollary.

We now specify the construction of the process $\tilde{{\textbf{X}}}(t)$, $t\in {\mathbb {R}}_+$, by specifying the initial condition and the updates. The initial condition $\tilde{{\textbf{X}}}(0)$ is specified as follows: each node *v* which is initialised with the best option 1 in the original system, ${\textbf{X}}$, is also initialised with option 1 in the coupled system, $\tilde{{\textbf{X}}}$. Each node which starts with any other option in the original system starts with option 2, the second-best option, in the coupled system. Nodes in the coupled system will only have preferences for options 1 or 2 at any time. Observe that the initialisation ensures that the condition in eqn. 16 holds at time $t=0$. We shall now construct the coupling in such a way that, if the condition holds just before an update, then it also holds just after the update. Hence, by induction, it holds at all times.

We now describe the joint evolution of both systems on a common probability space. We will explain in detail what happens at the first update time after time 0; the same reasoning applies to subsequent update times. Given the initial condition, the first update time at node *u* has an exponential distribution with parameter $\lambda _{X_u(0)}$ in the original system and $\lambda _{\tilde{X}_u(0)}$ in the coupled system. Let $\lambda _u=\max \{\lambda _{X_u(0)}, \lambda _{\tilde{X}_u(0)} \}$. We generate independent random variables $T_u\sim Exp(\lambda _u)$ for each $u\in V$, and set the first update time in the system to be $\min _{u\in V} T_u$. Suppose the minimum is attained at node $v$. At time $T_v$, we update the opinion of node $v$ in the original (resp. coupled) system with probability $\lambda _{X_v(0)}/\lambda _v$ (resp. $\lambda _{\tilde{X}_v(0)}/\lambda _v$). With the residual probability, there is no update. The update happens by node $v$ sampling one of its neighbours *w* chosen at random and copying its opinion; if both the original and coupled systems update at time $T_v$, then they both copy the same node *w*.

By the Markov property, we only need to specify what happens at the first update time (whether it occurs in the original system, the coupled system or both). The evolution after this time can be considered afresh starting from the state after the update. Hence, we only need to check that the condition in eqn. 16 is satisfied immediately after the update. We now check this for all possible scenarios.

Suppose first that $X_v(0)=\tilde{X}_v(0)=1$. Then $\lambda _v=\lambda _{X_v(0)}=\lambda _{\tilde{X}_v(0)}=1$, and node $v$ updates at time $T_v$ with probability 1 in both the original and coupled systems. As it updates by the copying the same node *w* in both systems, the opinion of node $v$ is the same in both systems after updating. Hence, condition 16 continues to be satisfied after the update.

Suppose next that $X_v(0)=j\ne 1$. Then, we must have $\tilde{X}_v(0)=2$; in the coupled system, nodes can only have opinions 1 or 2 and, by condition 16, it cannot be the case that $\tilde{X}_v(0)=1$. Hence, $\lambda _v=\max \{ \lambda _2,\lambda _j \}= \lambda _j$, as the $\lambda$’s have been sorted in increasing order (and site qualities in decreasing order), and $j\ge 2$. Hence, at time $T_v$, node *v* updates for sure in the original system, and with probability $\lambda _2/\lambda _j \le 1$ in the coupled system. If node $v$ updates in both systems, it does so by copying the same node *w*; hence, it has the same opinion in both systems are updating, so condition 16 continues to hold. If it updates in the original system but not the coupled system, then $\tilde{X}_v(t)$ remains unchanged and equal to 2 after the update. Hence, condition 16 continues to hold immediately after the update, no matter what value $X_v(t)$ takes.

Suppose, finally, that $X_v(0)=1$ but $\tilde{X}_v(0)=2$. (In fact, this cannot happen at the start, but can happen at some later update epoch and is hence a scenario that we need to consider.) Then, node $v$ updates for sure in the coupled system, and updates with probability $\lambda _1/\lambda _2<1$ in the original system. If it updates in both systems, it does so by copying the same node *w* and adopting the same opinion; hence, condition 16 continues to hold in this case. If it updates in the coupled system but not in the original system, then $X_v(t)$ remains equal to 1 in the original system. Hence, condition 16 holds after the update, irrespective of $\tilde{X}_v(t)$, the value adopted by $v$ in the coupled system after the update.

Thus, we have checked in all possible scenarios that, if condition 16 holds just before an opinion update at some node, then it continues to hold after the update. Hence, by induction, if this condition holds initially, then it holds for all time. This completes the proof of the corollary. $\square$

## Appendix B: Consensus times on regular graphs

### Proof of Theorem 2

Let $B(t)=|\{ v\in V:X_v(t)=B \}|$ denote the number of nodes holding opinion *B* at time *t*. On the complete graph, *B*(*t*) is a Markov process since it does not matter which particular nodes hold these opinions - all nodes are statistically identical. If $B(t)=m$, then there are $m(N-m)$ edges between nodes whose opinions differ. Along each of these edges, the *A* node contacts the *B* node at rate $1/(N-1)$ and changes its opinion to *B*, while the *B* node contacts the *A* node at rate $\lambda /(N-1)$ and changes its opinion to *A*. Hence, the Markov process *B*(*t*) has transition rates

$$\begin{aligned} \frac{m(N-m)}{N-1}: \; m \rightarrow m+1, \quad \frac{\lambda m(N-m)}{N-1}: \; m \rightarrow m-1, \end{aligned}$$

for $1\le m \le N-1$, and absorbing states at 0 and *N*.

As the total jump rate out of state *m* is $(1+\lambda )m(N-m)/(N-1)$, the Markov process *B*(*t*) spends an exponentially distributed random time with this parameter in state *m* before jumping to state $m+1$ with probability $\frac{1}{1+\lambda }$ or state $m-1$ with probability $\frac{\lambda }{(1+\lambda )}$, as noted in eqn. (11). Let $\tau _m$ denote the random time spent by *B*(*t*) in state *m* before jumping to state $m-1$ or $m+1$. Then, for $1\le m\le N-1$,

17 $$\begin{aligned} \begin{aligned} \tau _m&\sim Exp\Bigl ( \frac{(1+\lambda )m(N-m)}{N-1} \Bigr ), \\ \quad {\mathbb {E}}[\tau _m]&= \frac{N-1}{(1+\lambda )m(N-m)} = \frac{N-1}{n(1+\lambda )} \Bigl ( \frac{1}{m} + \frac{1}{N-m} \Bigr ), \end{aligned} \end{aligned}$$

where we use $\sim$ to denote equality in distribution.

Observe that the discrete-time process obtained by embedding *B*(*t*) at its jump times is an asymmetric random walk on $\{ 0,1,\ldots , N\}$, with absorbing boundaries at 0 and *N*. By this, we mean that the only possible transitions from a state $B(t)=m$ are to the states $m+1$ or $m-1$, and that the transition probabilities from *m* to $m+1$ do not depend on *m* (except at the boundaries) and likewise from *m* to $m-1$. The random walk is asymmetric because the transition probability, $\frac{1}{1+\lambda }$ from *m* to $m+1$ is different from the transition probability, $\frac{\lambda }{1+\lambda }$, from *m* to $m-1$. Let $C_j$ denote the random number of visits of this random walk to state *j* before absorption at 0 or *N*. Notice from the definition of the time to consensus *T* in (4) that the event ${\textbf{X}}(t)\equiv A$ corresponds to $B(t)=0$ and the event ${\textbf{X}}(t)\equiv B$ to $B(t)=N$. Hence, the time to consensus is the same as the time to absorption of the Markov process *B*(*t*), and we can express the expected time to consensus from an initial condition in which *k* nodes prefer Option *B* as follows:

18 $$\begin{aligned} {\mathbb {E}}[T|B(0)=k] = \sum _{j=1}^{N-1} {\mathbb {E}}[\tau _j] {\mathbb {E}}[C_j|B(0)=k], \end{aligned}$$

where ${\mathbb {E}}[\tau _j]$ is given in (17). The above equation decomposes the time up to absorption by the time spent in each of the intermediate states; in turn, this time can be split into the number of visits to each intermediate state and the time spent in that state during each visit. Finally, in taking expectations, we use the linearity of expectation, which says that the expectation of a sum of random variables is the sum of their expectations, and the independence between the number of visits to a state and the amount of time spent there on each visit, which is a consequence of the Markov property.

Thus, we can bound the mean time to consensus if we can bound the number of visits, $C_j$, to each state *j* for the asymmetric random walk on $\{ 0,1,\ldots ,N \}$. This is easy to do. Let ${\hat{C}}_j$ denote the number of visits to state *j* by the asymmetric random walk on the integer lattice ${\mathbb {Z}}$ with transition probabilities $\frac{1}{1+\lambda }$ from *j* to $j+1$ and $\frac{\lambda }{1+\lambda }$ from *j* to $j-1$, where $\lambda >1$. Clearly, $C_j$ is bounded above by ${\hat{C}}_j$. Moreover,

19 $$\begin{aligned} {\mathbb {E}}[{\hat{C}}_j|B(0)=k] = {\mathbb {P}}(\text{ hit } \text{ j }|B(0)=k)\frac{1}{1-p} \le \frac{1}{1-p}, \end{aligned}$$

where $p<1$ is the probability that the asymmetric random walk on the integers ever returns to state *j*; it does not depend on *j*, by translation invariance. An easy calculation shows that $p=2/(1+\lambda )$. Hence, we obtain from (17) and (18) that

$$\begin{aligned} {\mathbb {E}}[T|B(0)=k] \le \frac{N-1}{N(1+\lambda )} \frac{1+\lambda }{\lambda -1} \sum _{j=1}^{N-1} \Bigl ( \frac{1}{j}+\frac{1}{N-j} \Bigr ) \le \frac{2(1+\log N)}{\lambda -1}, \end{aligned}$$

where the second inequality follows from the fact that

$$\begin{aligned} \sum _{j=1}^{N-1} \frac{1}{N-j} = \sum _{j=1}^{N-1}\frac{1}{j} \le 1+\int _1^N \frac{1}{x}dx = 1+\log N. \end{aligned}$$

This yields the claim of the theorem for complete graphs.

The proof for regular graphs is quite similar; the discrete-time process counting the number of nodes with opinion *B* evolves as an asymmetric random walk with the same transition probabilities as for the complete graph. The difference is in the time between jumps of the process *B*(*t*).

Let ${\textbf{X}}(t)$ denote the configuration at time *t* and define $S_t=\{ v\in V: X-v(t)=B \}$ to be the set of nodes with opinion *B*. The total rate at which a node in $S_t$ contacts a node in $S_t^c$, and hence changes its opinion from *B* to *A*, is $\lambda |E(S_t,S_t^c)|/d$. Similarly, the total rate of contacts from $S_t^c$ to $S_t$, which result in one node changing opinion from *A* to *B*, is $|E(S_t,S_t^c)|/d$. Thus, the total jump rate out of the state $B(t)=m$ is $\frac{\lambda +1}{d}|E(S_t,S_t^c)|$. Notice that this depends not only on *m* but also on the set $S_t$, which is determined by the configuration ${\textbf{X}}(t)$. Denoting the time spent in state $B(t)=m$ by $\tau _m$, we thus get

$$\begin{aligned} {\mathbb {E}}[\tau _m|{\textbf{X}}(t), B(t)=m] = \frac{d}{\lambda +1} \frac{1}{|E(S_t,S_t^c)|}. \end{aligned}$$

Now, observe from the definition of the isoperimetric constant $\eta$ that

$$\begin{aligned} |E(S_t, S_t^c)| \ge \eta \min \{ |S_t|,|S_t^c| \} \ge \frac{\eta }{n} |S_t| \, |S_t^c|. \end{aligned}$$

But $|S_t|=m$ and $|S_t^c|=N-m$ if $B(t)=m$. Hence, we obtain from the above that

20 $$\begin{aligned} {\mathbb {E}}[\tau _m| {\textbf{X}}(t), B(t)=m] \le \frac{d}{\eta (\lambda +1)} \frac{n}{m(N-m)}. \end{aligned}$$

The rest of the calculations are exactly the same as for the complete graph, since the expected number of visits to each state $B(t)=m$ before absorption is the same. Comparing the expressions in (17) and (20), we see that the time spent in the state $B(t)=m$ on each visit is at most $d/\eta$ times as large on a *d*-regular graph with isoperimetric constant $\eta$ as it is on the complete graph. Hence, the time to consensus is also larger by at most this same factor. This completes the proof of the theorem. $\square$

## Appendix C: Robustness of consensus probabilities and times

### Proof of Theorem 3

We omit calculations which are very similar to those in the proofs of the two theorems above, and only emphasise what is different.

Let ${\textbf{X}}(t)$ denote the vector of agent opinions at time $t\in {\mathbb {R}}_+$ and ${\textbf{Y}}(t)$, $t\in {\mathbb {N}},$ the embedded discrete-time process at node update times, as defined earlier. Then, ${\textbf{X}}$ and ${\textbf{Y}}$ are Markov processes in continuous and discrete time respectively.

Let *B*(*t*) denote the number of agents which prefer option *B* at time $t\in {\mathbb {N}}$. First, we observe that $M(t)=\mu ^{B(t)}$ is a supermartingale, i.e.,

$$\begin{aligned} {\mathbb {E}}[M(t+1)|{\textbf{Y}}(s), s\le t] \le M(t). \end{aligned}$$

Define *T* to be the time to reach consensus, and note that it is a stopping time which is finite with probability 1. Moreover, *M*(*t*) is bounded. Hence, by the Optional Stopping Theorem, ${\mathbb {E}}[M(T)] \le M(0)$. In other words,

$$\begin{aligned} {\mathbb {P}}(Y(T)\equiv A)+\mu ^N {\mathbb {P}}(Y(T) \equiv B) \le \mu ^{N-k}, \end{aligned}$$

since $B(0)=N-k$, by assumption. The first claim of the theorem follows by re-arranging the above.

Let ${\textbf{X}}(t)$ be a configuration with $B(t)=m$ nodes preferring option *B*, and let $S_t$ denote the set of these nodes. The total rate at which nodes in $S_t$ contact nodes in $S_t^c$ and switch opinion to *A* is given by

21 $$\begin{aligned} \sum _{v\in S_t} \frac{\lambda }{deg(v)} |\{ w\in S_t^c: (v,w)\in E \}| \ge \frac{\lambda }{d_{\max }} \sum _{v\in S_t} |\{ w\in S_t^c: (v,w)\in E\}| = \frac{\lambda }{d_{\max }} |E(S_t,S_t^c)|. \end{aligned}$$

Similarly, the total rate at which nodes in $S_t^c$ contact nodes in *A* and switch opinion to *B* is given by

22 $$\begin{aligned} \sum _{v\in S_t^c} \frac{1}{deg(v)} |\{ w\in S_t: (v,w)\in E \}| \le \frac{1}{d_{\min }} \sum _{v\in S_t^c} |\{ w\in S_t: (v,w)\in E\}| = \frac{1}{d_{\min }} |E(S_t,S_t^c)|. \end{aligned}$$

It follows that

23 $$\begin{aligned} \begin{aligned}&{\mathbb {P}}(B(t+1)=m+1|B(t)=m,{\textbf{X}}(t)) \le \frac{1/d_{\min }}{(1/d_{\min })+(\lambda /d_{\max })} = \frac{1}{\mu +1}, \\&{\mathbb {P}}(B(t+1)=m-1|B(t)=m,{\textbf{X}}(t)) \ge \frac{\mu }{\mu +1}. \end{aligned} \end{aligned}$$

Let *Z*(*t*) denote the asymmetric random walk on the integers with probability $\frac{1}{\mu +1}$ of going from *m* to $m+1$ and probability $\frac{\mu }{\mu +1}$ of going from *m* to $m-1$. Then, from any state *m*, *Z*(*t*) has at least as great a chance as *B*(*t*) of moving to $m+1$, and at most as great a chance of moving to $m-1$. Hence, we can couple these two processes (define them on the same probability space) in such a way that, if they start in the same initial condition $Z(0)=B(0)=j$, then $Z(t)\ge B(t)$ for all $t\le T$, where *T* denotes the absorption time of *B*(*t*) at either 0 or *n*. It follows from this that

$$\begin{aligned} {\mathbb {P}}(\exists 1\le t\le T: B(t)=j) \le {\mathbb {P}}(\exists t\ge 1: Z(t)=j), \end{aligned}$$

i.e., the probability that the $Z(\cdot )$ process ever returns to *j* is at least as large as for the $B(\cdot )$ process. To see this, note that if $B(1)=j+1$, then $Z(1)=j+1$ as well (since $Z(1)\ge B(1)$ by the coupling), and there is guaranteed to be a $t\ge 1$ such that $Z(t)=j$, since the random walk $Z(\cdot )$ has negative drift and eventually tends to $-\infty$. Suppose on the other hand that $B(1)=j-1$. If $Z(1)=j+1$, then we noted that the $Z(\cdot )$ process is guaranteed to return to *j*. Finally, if $Z(1)=j-1$ and there is a $t\ge 2$ at which *B*(*t*) returns to *j*, then $Z(t)\ge j$. But if $Z(\cdot )$ went from $j-1$ at time 1 to some $k\ge j$ at time *t*, it must have passed through *j* before *t* or be in *j* at time *t*.

If the return probability to any state *j* is at least as large for the $Z(\cdot )$ process, then the expected number of returns to *j* is also at least as large. Thus, we can use the same approach as in the proof of Theorem 2 to bound the consensus time by bounding the number of visits of the $B(\cdot )$ process to any state *j*, and the amount of time spent in state *j* during each visit. We have bounded the number of visits by the number of returns to any state of an asymmetric random walk; the only difference from the corresponding random walk in the proof of Theorem 2 is that $\lambda$ has been replaced with $\mu$.

We now turn to the residence time in state *j*. Let ${\textbf{X}}(t)$ be a configuration in which $B(t)=j$ and let $S_t$ denote the set of nodes with opinion *B*. We obtained an upper bound on the jump rate of $B(\cdot )$ from *j* to $j+1$ in (22), but can also obtain a lower bound. The total rate at which nodes in $S_t^c$ contact nodes in $S_t$ and change opinion from *A* to *B* is given by

24 $$\begin{aligned} \sum _{v\in S_t^c} \frac{1}{deg(v)} |\{ w\in S_t:(v,w)\in E \}| \ge \frac{1}{d_{\max }} |E(S_t,S_t^c)|. \end{aligned}$$

Combining (21) and (24), the total jump rate out of state $B(t)=j$ is at least $\frac{\lambda +1}{d_{\max }}|E(S_t,S_t^c)|$. Hence, the mean time spent in a state *j* during any single visit is bounded above as

$$\begin{aligned} {\mathbb {E}}[\tau _j] \le \frac{d_{\max }}{(\lambda +1)|E(S_t,S_t^c)|} \le \frac{d_{\max }}{(\lambda +1)\eta } \frac{N}{j(N-j)}, \end{aligned}$$

which is the same expression obtained during the proof of Theorem 2 except that *d* has been replaced by $d_{\max }$. Following the same calculations as in that proof completes the proof of the theorem. $\square$

## Appendix D: Comparison of extinction probabilities

### Proof of Theorem 6

Define $g_i:(0,\infty ) \rightarrow (0,\infty )$ by $g_i(c)={\mathbb {E}}[\lambda /(\lambda c+R_A^i)]$ for $i=1,2$. We have from Theorem 5 that $\pi ^i=1-c^i$, where $c^i$ is the unique positive solution of $g^i(c)=1$. We can rewrite $g_i$ in terms of $T_A^i=1/R_A^i$ as follows:

$$\begin{aligned} g_i(c) = {\mathbb {E}}\Bigl [ \frac{\lambda T_A^i}{\lambda c T_A^i+1} \Bigr ] = \frac{1}{c} \Bigl ( 1-{\mathbb {E}}\Bigl [ \frac{1}{\lambda c T_A^i+1} \Bigr ] \Bigr ). \end{aligned}$$

Observe that $t\mapsto \frac{1}{1+\lambda ct}$ is a convex function for any $c>0$. Hence, if ${{\hat{F}}}_A^1 {\preceq }_{cx}{{\hat{F}}}_A^2$, then

$$\begin{aligned} 1 = g_1(c^1) = \frac{1}{c^1} \Bigl ( 1-{\mathbb {E}}\Bigl [ \frac{1}{\lambda c^1 T_A^1+1} \Bigr ] \Bigr ) \ge \frac{1}{c^1} \Bigl ( 1-{\mathbb {E}}\Bigl [ \frac{1}{\lambda c^1 T_A^2+1} \Bigr ] \Bigr ) = g_2(c^1). \end{aligned}$$

As $g_2$ is clearly a decreasing function, it follows that $c^2$, the solution of $g_2(c)=1$, must satisfy $c^2\le c^1$. Since $\pi ^i=1-c^i$, we have $\pi ^1 \le \pi ^2$, as claimed. $\square$

## Footnotes

1 The Moran model in population genetics describes the evolution of the number of alleles of a given type in a population of *N* genes. Each gene may be of one of *n* types, or alleles; say $n=2$, for simplicity, and denote the alleles by *A* and *B*. The population size, *N*, stays constant. Each copy of allele *A* lives for a random time with an $Exp(\lambda _A)$ distribution, and each copy of allele *B* for an $Exp(\lambda _B)$ random time. Lifetimes of distinct copies are mutually independent. When an allele dies, it is instantaneously replaced by a copy of another allele chosen uniformly at random from the population. If $\lambda _A=\lambda _B$, it is known as the neutral Moran model, whereas if they are different, it is called the Moran model with selection. The reciprocal of $\lambda _A$, which is the mean lifetime of allele *A*, is termed its fitness. Note that this is exactly equivalent to the Weighted Voter Model.

2 There is a substantial literature in distributed computing on Byzantine fault tolerance, which allows for agents to be malicious or adversarial. Faulty robots are studied in Crosscombe et al. (2017), for instance. While a detailed adversarial analysis of the Weighted Voter Model algorithm is outside the scope of this paper, we mention it here as a potential direction for future research.

3 A branching process is a random process model of an asexually reproducing population. In the simplest case, individuals live for a single generation of fixed duration, during which they have a random number of offspring according to a given distribution, independent of other individuals. They have been used to model bacteria, neutrons in a chain reaction, etc. In a multitype branching process, individuals of different types have offspring according to different distributions. These have been widely studied in probability theory. Our interest is in the probability that the population becomes extinct, which corresponds to consensus on the worse alternative.

4 Note that, throughout this section, *k* will refer to the number of agents initially preferring the higher quality site, even though we have used *l* elsewhere to denote this quantity. As numerical values often appear in this section, we wanted to avoid the potential for confusion between *l* and 1.
